# Extensive Proliferation of a Subset of Differentiated, yet Plastic, Medial Vascular Smooth Muscle Cells Contributes to Neointimal Formation in Mouse Injury and Atherosclerosis Models

**DOI:** 10.1161/CIRCRESAHA.116.309799

**Published:** 2016-09-28

**Authors:** Joel Chappell, Jennifer L. Harman, Vagheesh M. Narasimhan, Haixiang Yu, Kirsty Foote, Benjamin D. Simons, Martin R. Bennett, Helle F. Jørgensen

**Affiliations:** From the Cardiovascular Medicine Division, Department of Medicine (J.C., J.L.H., H.Y., K.F., M.R.B., H.F.J.), Cavendish Laboratory, Department of Physics (B.D.S.), The Wellcome Trust/Cancer Research UK Gurdon Institute (B.D.S.), and Wellcome Trust-Medical Research Council Stem Cell Institute (B.D.S.), University of Cambridge, United Kingdom; and The Wellcome Trust Sanger Institute, Hinxton, Cambridge, United Kingdom (V.M.N.).

**Keywords:** atherosclerosis, lineage-tracing, macrophages, neointima, phenotype, vascular diseases vascular smooth muscle

## Abstract

**Rationale:**

Vascular smooth muscle cell (VSMC) accumulation is a hallmark of atherosclerosis and vascular injury. However, fundamental aspects of proliferation and the phenotypic changes within individual VSMCs, which underlie vascular disease, remain unresolved. In particular, it is not known whether all VSMCs proliferate and display plasticity or whether individual cells can switch to multiple phenotypes.

**Objective:**

To assess whether proliferation and plasticity in disease is a general characteristic of VSMCs or a feature of a subset of cells.

**Methods and Results:**

Using multicolor lineage labeling, we demonstrate that VSMCs in injury-induced neointimal lesions and in atherosclerotic plaques are oligoclonal, derived from few expanding cells. Lineage tracing also revealed that the progeny of individual VSMCs contributes to both alpha smooth muscle actin (aSma)–positive fibrous cap and Mac3-expressing macrophage-like plaque core cells. Costaining for phenotypic markers further identified a double-positive aSma+ Mac3+ cell population, which is specific to VSMC-derived plaque cells. In contrast, VSMC-derived cells generating the neointima after vascular injury generally retained the expression of VSMC markers and the upregulation of Mac3 was less pronounced. Monochromatic regions in atherosclerotic plaques and injury-induced neointima did not contain VSMC-derived cells expressing a different fluorescent reporter protein, suggesting that proliferation-independent VSMC migration does not make a major contribution to VSMC accumulation in vascular disease.

**Conclusions:**

We demonstrate that extensive proliferation of a low proportion of highly plastic VSMCs results in the observed VSMC accumulation after injury and in atherosclerotic plaques. Therapeutic targeting of these hyperproliferating VSMCs might effectively reduce vascular disease without affecting vascular integrity.

## Abstract 2

Supplemental Digital Content is available in the text.

Vascular smooth muscle cell (VSMC) accumulation is a hallmark of atherosclerosis,^1^ and VSMCs also generate the bulk of the neointima formed after vessel occlusion or injury.^2–4^ VSMCs display remarkable phenotypic plasticity in vitro,^5^ and lineage-tracing experiments have convincingly shown that VSMC phenotypic switching occurs in vivo.^3,4,6–9^ Healthy, mature VSMCs are quiescent and express contractile genes, such as alpha smooth muscle actin (aSma) and smooth muscle myosin heavy chain (SMMHC/Myh11). These contractile genes are downregulated when VSMCs undergo phenotypic switching, which result in increased proliferation, migration, and expression of extracellular matrix.^10^ Lineage-tracing experiments have demonstrated that VSMC-derived cells not only form the aSma-positive cells in the fibrous cap, which protects from plaque rupture, but also contribute substantially to the generation of the plaque core. Specifically, mouse models of atherosclerosis manifest VSMC-derived cells that lack aSma and the mature VSMC marker SMMHC/Myh11 but instead express genes associated with other cell types, including macrophages (Mac3),^6–9^ which may contribute negatively to disease progression. Importantly, this transdifferentiation is also observed in human plaques,^6,8^ highlighting the importance of VSMC plasticity in disease.

**Editorial, see p 1262**

**In This Issue, see p 1255**

VSMC migration has also been proposed to play a major contribution to the accumulation of VSMCs in disease, with suggestions that up to 50% of neointimal cells result from migration of nondividing cells after vascular injury.^11,12^ Despite extensive investigation of regulators of VSMC phenotypic switching, the proliferation, migration, and plasticity of individual VSMCs in vascular disease, however, remain controversial. In particular, it is unknown whether all, or only a fraction, of VSMCs in major arteries proliferate and display plasticity and whether individual cells can switch to multiple phenotypes or whether these arise from different VSMC subsets.

Here, we report that clonal expansion of a low proportion of mature VSMCs underlies VSMC accumulation after vascular injury and demonstrate that individual VSMCs have the ability to generate plaque cells of different phenotypes. Furthermore, we find that VSMCs within the media underlying atherosclerotic plaques display features of phenotypic switching without contributing to cells within the plaque. Importantly, our data strongly suggest that migration of nonproliferating cells makes a minor contribution to VSMC accumulation in disease.

## Methods

Detailed descriptions of experimental procedures and animals used are provided in the Online Data Supplement.

All animal experiments were approved by the UK Home Office (PPL70/7565) and the local ethics committee and were performed according to the UK Home Office guidelines. The Myh11-CreERt2,^13^ Rosa26-Confetti,^14^ and ApoE^−^^/−^^15^ lines have all been described. For atherosclerosis studies, Myh11-CreERt2/Rosa26-Confetti/ApoE^−/−^ animals were injected with tamoxifen at 6 to 8 weeks and were fed a high-fat diet from week 9 until analysis, as described in Online Data Supplement. Tissue was sectioned, immunostained, and cleared before the confocal microscopy analysis as described in the Online Data Supplement. Animals (Myh11-CreERt2/Rosa26-Confetti) undergoing carotid ligation surgery were injected with tamoxifen at 6 to 8 weeks, the left common carotid artery ligated at the bifurcation 1 week after the final tamoxifen injection, and allowed to recover for 28 days post surgery. Whole-mounted tissue was cleared and analyzed by confocal microscopy, followed by cryosectioning, immunostaining, and clearing before being reanalyzed by confocal microscopy.

Confocal microscopy Z-stacks were processed using Imaris software (Bitplane, Zurich, Switzerland). Images displayed are maximal projections of confocal Z-stacks (generated in Imaris) where indicated in figure legends or individual scans (generated in FIJI). Quantification was performed on confocal Z-stacks in Imaris as described in the Online Data Supplement to ensure correct scoring of staining in individual cells.

## Results

### Multicolor Labeling of VSMCs

**Figure 1.**
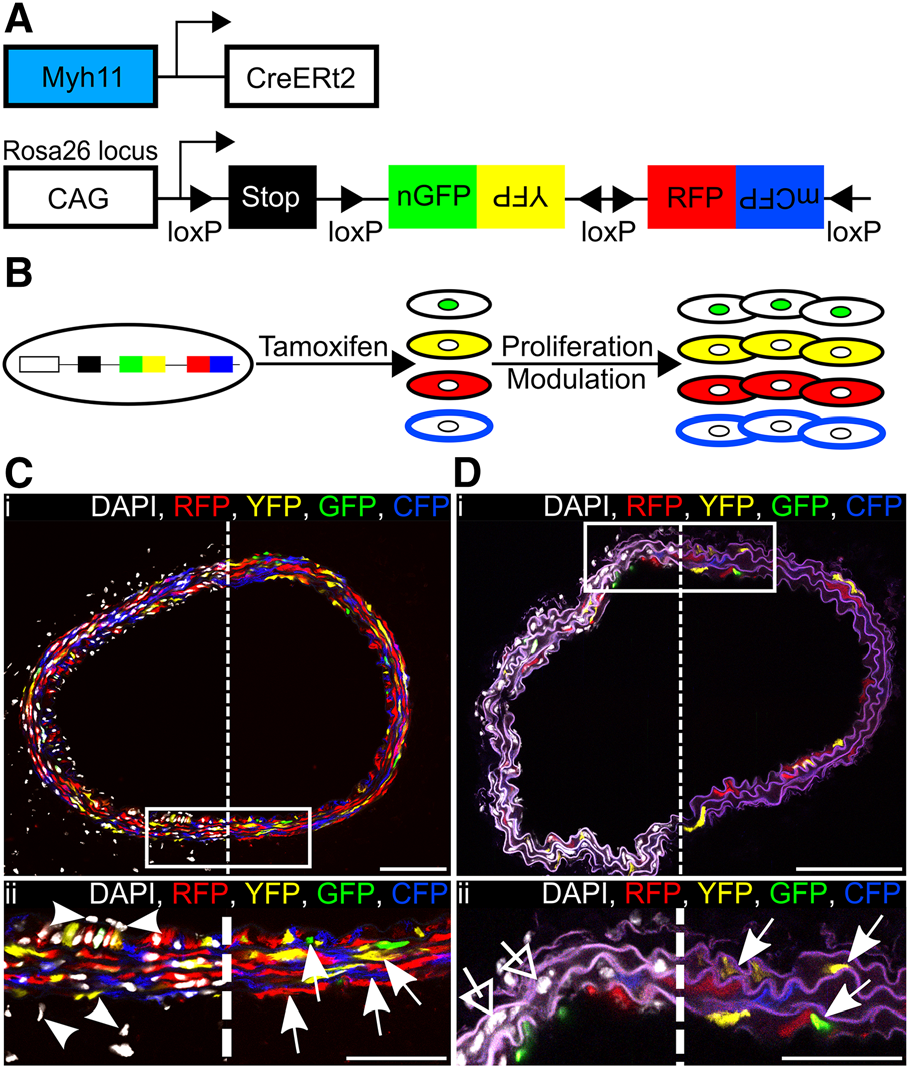
**Efficient and specific multicolor vascular smooth muscle cell (VSMC) labeling in Myh11-CreERt2/Rosa26-Confetti animals. A**, Schematic representation of the Myh11-CreERt2 transgene and the Rosa26-Confetti reporter allele. **B**, Schematic representation illustrating tamoxifen-induced recombination at the Rosa26-Confetti locus, resulting in expression of 1 of 4 fluorescent proteins, which are stably propagated independent of Myh11 expression within progeny. **C** and **D**, Carotid artery cross sections from high density–labeled (**C**; 10× 1 mg tamoxifen) or low density–labeled (**D**; 1× 0.1 mg tamoxifen) animals; region outlined in (i) is magnified in (ii). Signals for fluorescent proteins are shown with (left) and without (right) nuclear DAPI (4',6-diamidino-2-phenylindole) staining (white). **C**, VSMCs, indicated by arrows in (ii), are labeled with red fluorescent protein (RFP), yellow fluorescent protein (YFP), nuclear (n) green fluorescent protein (GFP), or membrane-associated (m) cyan fluorescent protein (CFP), whereas cells within the adventitia and endothelium, indicated by arrow heads, are unlabeled. In (**D**[ii]), arrows point to the few labeled VSMCs, and open arrows point to unlabeled VSMCs within the media. Scale bars are 100 μm in (i) and 50 μm in (ii).

To study the proliferation of individual VSMCs in vivo, we combined the inducible Myh11-CreERt2 transgene,^13^ which is specifically expressed in mature VSMCs, with the Rosa26-Confetti multicolor reporter allele (Figure 1A).^14^ We induced recombination in healthy animals with a pulse of tamoxifen, which resulted in random labeling of 70% to 95% of VSMCs with 1 of 4 fluorescent proteins (Online Table I). There was a slight bias toward recombination events resulting in expression of red fluorescent protein in 36% of labeled cells, compared with 26% for yellow fluorescent protein, 9% for nuclear green fluorescent protein, and 29% for membrane-bound cyan fluorescent protein (Online Figure I, light gray bars). Importantly, the specific Confetti label is stably transferred to all progeny after VSMC proliferation independent of expression status of the Myh11-CreERt2 transgene and will, therefore, remain expressed after phenotypic switching (Figure 1B). As expected for a tissue with a low proliferative index, the stochastic labeling of elongated VSMCs generated a mosaic pattern (Figure 1C; Online Figure II), which was constant over months in healthy animals. Recombination was specific to VSMCs as demonstrated by the lack of labeling of cells in other tissues, including adventitia (Online Figure II), bone marrow, peripheral blood, skeletal muscle, lung, and liver (Online Figure III), similar to what was reported in published studies using this transgene with other reporter alleles.^6,8,9,13^

### Clonal Expansion of Few VSMCs in Atherosclerotic Plaques

**Figure 2.**
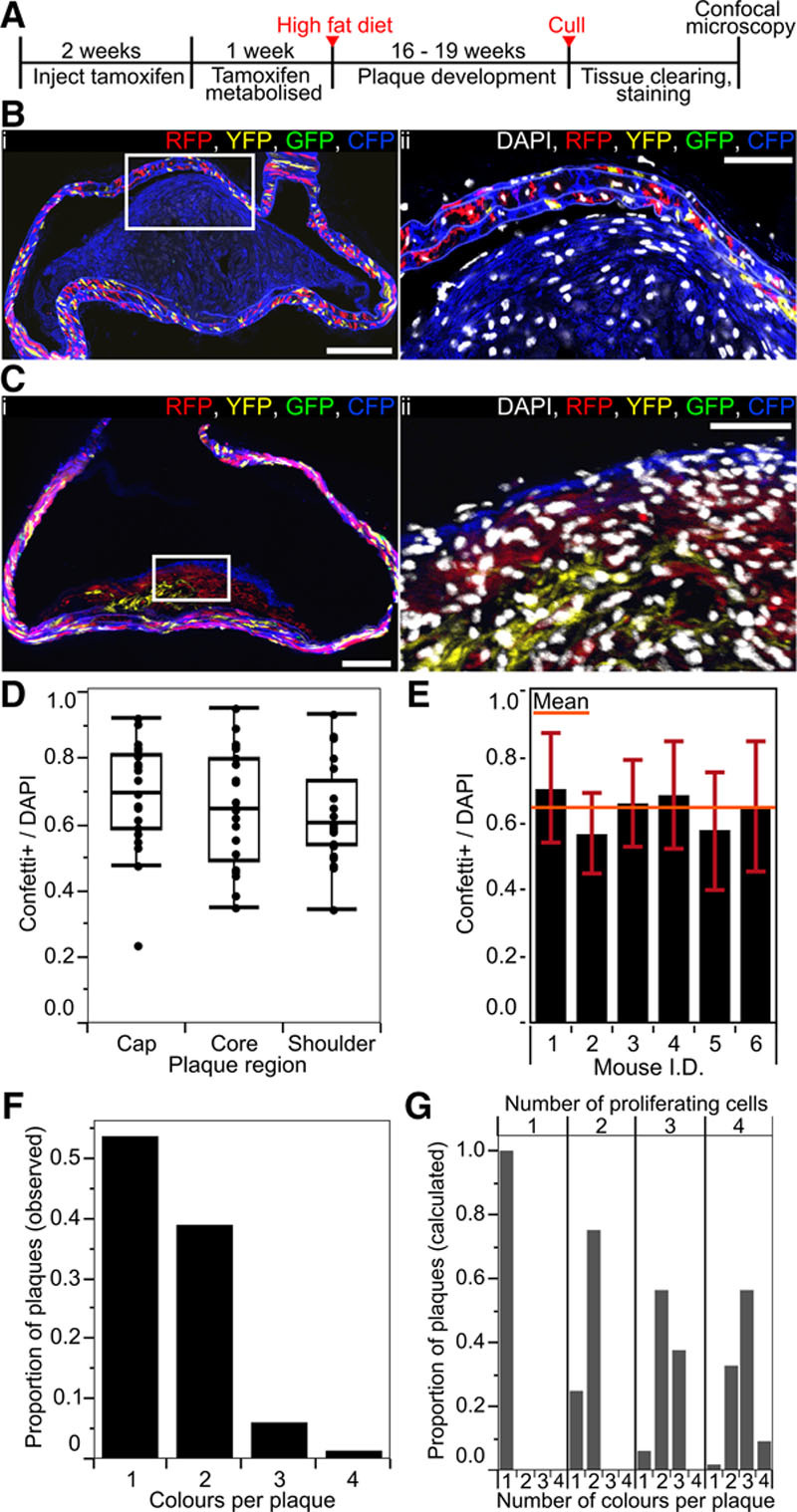
**Vascular smooth muscle cell (VSMC)–derived cells generate oligoclonal atherosclerotic plaques. A**, Experimental protocol for the atherosclerosis studies. **B** and **C**, Arterial cryosections from high density–labeled animals (10× 1 mg tamoxifen) presenting plaques containing VSMC-derived cells of a single color (membrane-associated cyan fluorescent protein [CFP]; **B**) and >1 color (red fluorescent protein [RFP], membrane-associated CFP, and yellow fluorescent protein [YFP]; **C**). The region outlined in (i) is magnified in (ii). Scale bars are 150 μm (i) and 50 μm (ii). Signals for fluorescent proteins and nuclear DAPI (4',6-diamidino-2-phenylindole; in ii, white) are shown. **D**, Box plot showing the proportion of the total number of cells (DAPI), which express the Confetti reporter (Confetti+) within each plaque region (in 23 plaques from 6 animals). **E**, Bar chart showing the proportion of the total number of cells (DAPI), which express the Confetti reporter (Confetti+) within plaques for 6 individual animals (23 plaques from 6 animals). Mean across all animals is indicated by the orange bar, SD±0.16. **F**, Bar chart showing the number of colors observed per plaque (82 plaques from 16 animals). **G**, Bar chart showing the theoretical distribution of colors per plaque resulting from proliferation of 1, 2, 3, or 4 VSMCs labeled at the recombination frequency observed in high density–labeled animals. All data are from animals labeled at high density (10× 1 mg tamoxifen).

To study clonal VSMC proliferation in disease, we crossed Myh11-CreERt2/Rosa26-Confetti animals onto an ApoE^−^^/−^ background^15^ and induced recombination in 6- to 8-week-old animals before feeding them an atherosclerosis-inducing high-fat diet (21% fat and 0.2% cholesterol) for 16 to 19 weeks (Figure 2A; Online Table II). Accumulation of VSMC-derived cells was assessed by confocal microscopy of arterial cross sections. As expected,^6,8^ cells expressing the Confetti reporter, which are derived from Myh11-expressing VSMCs, contributed to both cap and core regions (Figure 2B and 2C; Online Figure IV) and constituted a significant proportion of the total cell number within atherosclerotic lesions. Interestingly, VSMC contribution was similar for the fibrous cap, shoulder, and core regions (Figure 2D). On average, 70% of plaque cells were label-positive (Confetti+) and hence derived from VSMCs, but this ranged from 40% to 90%. Stratification of VSMC contribution according to the vascular region (Online Figure VA) or individual animals (Figure 2E) did not reveal significant differences between regions, suggesting that interplaque variation is not linked to blood lipid level variation or reflects differences in hemodynamic or VSMC developmental origin.^16^

In contrast to the mosaic stochastic labeling observed in the vascular wall, VSMC-derived cells within plaques were found in large monochromatic regions with little intermixing between colors as shown in thick 50- to 100-μm sections (Online Figure VI; Online Movie I) or 20-μm cryosections (Figure 2B and 2C). Plaques showed substantial variation in color pattern (Online Figure VI), but the frequency of individual colors closely correlated with labeling frequency, demonstrating that there was no bias toward expansion of particular colors (Online Figure I). Quantification of colored regions revealed that most plaques contained 1 or 2 colors (52% and 40%) with a small proportion presenting 3 (6%) or 4 colors (2%; Figure 2F). We compared this observed distribution of colors per plaque (Figure 2F) to the expected distributions in plaques derived from the proliferation of 1, 2, 3, or 4 cells (Figure 2G), which were calculated based on the observed recombination frequency. The disparity from the expected color pattern generated if 3, 4, or more cells proliferate, strongly argues that, typically, a few mature Myh11-expressing VSMCs undergo massive expansion to contribute to plaque formation. However, our analysis clearly shows that plaques in atherosclerotic animals are not exclusively monoclonal, in agreement with recent studies using lineage tracing of SM22a-expressing cells,^7^ but in contrast to X-inactivation–based analysis of human lesions.^17^

We confirmed that monochromatic regions arise from clonal expansion of a single cell using lower tamoxifen doses to achieve reduced labeling frequencies. A 10-fold reduction in tamoxifen dose (to 1× 1 mg/animal) resulted in labeling 40% of VSMCs, which produced many unlabeled plaques or plaques, where a single-colored region occupied a large part of the plaque area (Online Figure VII). In addition, we only observed a single monochromatic region within plaques from 4 animals receiving 0.1 mg tamoxifen (Online Table II). Importantly, the size of the monochromatic regions observed in animals with reduced labeling frequency was comparable to what was found in high density–labeled animals (Online Figure VII). On the basis of these observations, we conclude that monochromatic regions within lesions are generally the progeny of a single cell, signifying that VSMC-derived cells in atherosclerotic lesions are typically formed by the clonal expansion of few VSMCs.

### Generation of Phenotypically Distinct Cells From VSMCs

**Figure 3.**
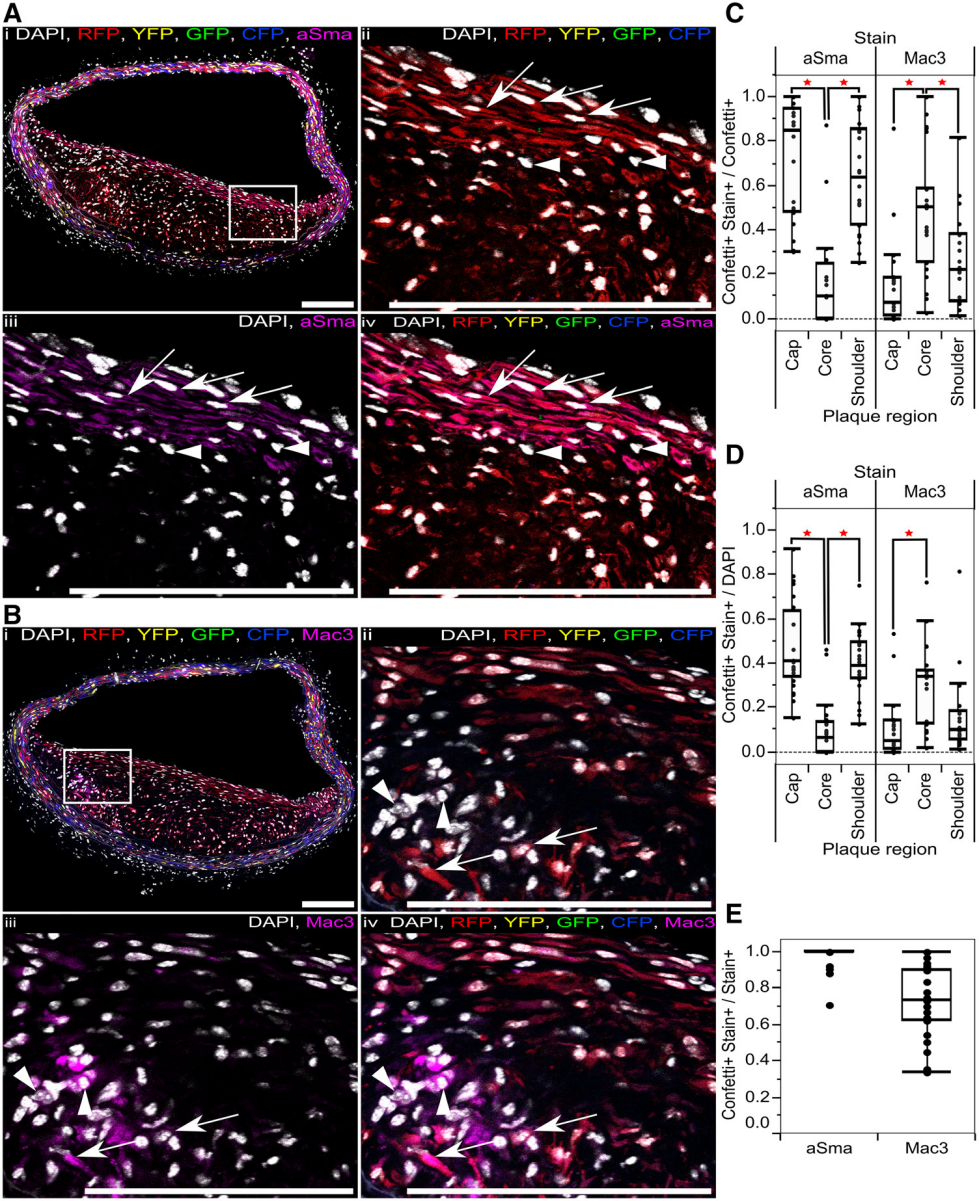
**Progeny of a single vascular smooth muscle cell (VSMC) can adopt multiple phenotypes in disease. A** and **B**, Immunostaining for alpha smooth muscle actin (aSma; **A**) and Mac3 (**B**) of serial cryosections from high density–labeled animal (10× 1 mg tamoxifen) containing RFP-expressing VSMC-derived cells. Signals for fluorescent proteins, nuclear DAPI (4',6-diamidino-2-phenylindole; white), aSma (magenta; **A**), and Mac3 (magenta; **B**) are shown as indicated on each image. Regions outlined in (i) are magnified in panels (ii through v), scale bars are 150 μm. **A**, Arrows point to RFP+ aSma+ cells, arrow heads point to RFP− aSma− cells. **B**, Arrows point to RFP+ Mac3+ cells, and arrow heads point to RFP− Mac3+ cells. **C through E**, Box plot showing proportion of cells that express the Confetti reporter and stain positive for either aSma or Mac3 (Confetti+ Stain+), relative to all cells expressing the Confetti reporter (Confetti+; **C**), the total number of cells (DAPI; **D**), or all cells staining positive for aSma or Mac3 markers (Stain+; **E**). A red star indicates a significant difference (*P*<0.05) determined by a 2-way analysis of variance. Data in (**C through E**) are from 23 plaques from 6 animals. All data are from animals labeled at high density (10× 1 mg tamoxifen). CFP indicates cyan fluorescent protein; GFP, green fluorescent protein; RFP, red fluorescent protein; and YFP, yellow fluorescent protein.

To assess VSMC plasticity and origin of plaque cells with different phenotypes within monochromatic plaque regions, we stained sequential cryosections for either the VSMC marker aSma or Mac3, which is upregulated as VSMCs adopt a macrophage-like cell type (Figure 3A and 3B). Quantification of staining patterns in different plaque regions showed that aSma+ cells were abundant in the cap and shoulder regions (82% and 62%, respectively, of cells expressing the Confetti reporter), whereas, on average, only 10% of VSMC-derived cells (8% of all cells) in the plaque core expressed aSma (Figure 3C and 3D). Two different single-color lineage-tracing studies reported that 18%^6^ or 70%^9^ of aSma-expressing cells were VSMC derived, which both lie within the range shown here. The variation possibly reflects the differences between plaque regions, which was not considered in these studies.^6,9^ We find that almost all (median 100% and mean 97%) aSma+ cells are derived from Myh11-expressing cells (Figure 3E). This is consistent with the observations that bone marrow–derived cells rarely upregulate VSMC markers, including aSma.^18^ The apparent discrepancy with a recent study showing a significant contribution of LysM-Cre-expressing cells to aSma+ plaque cells^9^ possibly results from the use of a constitutively active recombinase (rather than tamoxifen inducible) in that study, which might be induced in VSMC-derived plaque cells.

Mac3+ VSMC-derived cells were located predominantly in the core, where they comprised a significant proportion (median 50% of VSMC derived/Confetti+, 34% of all cells, and 73% of Mac3+ cells; Figure 3C through 3E). However, we also observed a variable, but significant number of Mac3-expressing VSMC-derived/Confetti+ cells in the cap and shoulder regions (7% and 22%, respectively). Interplaque variation in staining pattern was not explained by either vascular region or differences between animals, except for 1 mouse showing significantly lower VSMC contribution to Mac3-expressing cells (Online Figure VB, VC, and VIII). For comparison, previous studies observed that phenotypically modulated VSMCs generate ≈30% of all plaque cells^6^ and that 12% of plaque cells expressing another macrophage marker CD68^9^, which is consistent with our findings.

### Individual VSMCs Generate Cells Displaying Different Phenotypes

**Figure 4.**
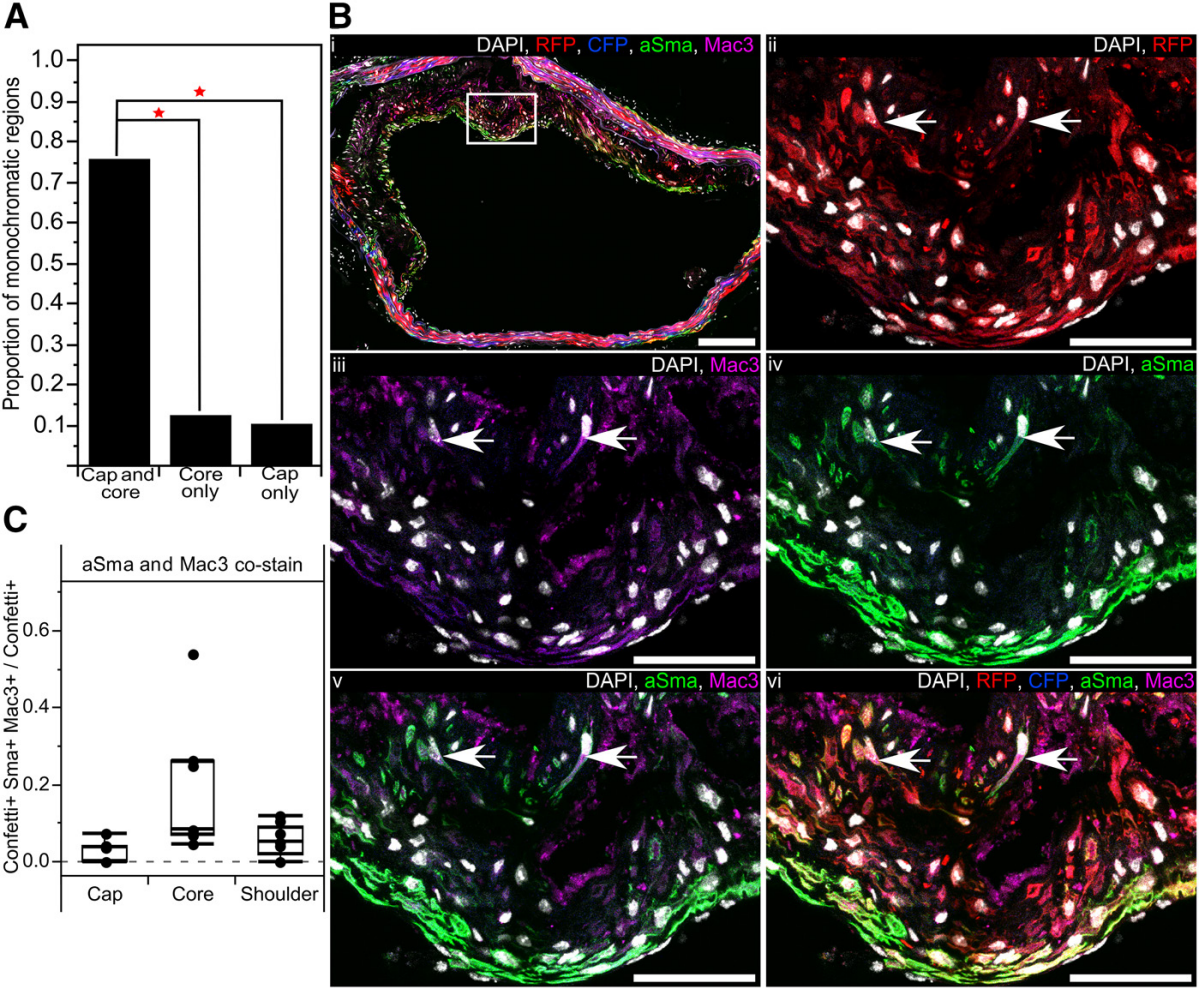
**Progeny of a single vascular smooth muscle cell (VSMC) generates plaque cells with different phenotypes. A**, Bar chart showing the proportion of monochromatic regions, which occupy both the cap and core or only a single region within an atherosclerotic plaque (82 plaques from 16 high density–labeled animals). **B**, Arterial cryosection from artery containing red fluorescent protein (RFP)–expressing VSMC-derived cells, costained for alpha smooth muscle actin (aSma) and Mac3. The region outlined in (i) is magnified in (ii through iv). Arrows point to RFP+ Mac3+ aSma+ cells. Scale bars are 150 μm (i) and 50 μm (ii through vi). Signals for fluorescent proteins, nuclear DAPI (4',6-diamidino-2-phenylindole; white), aSma (green) and Mac3 (magenta) are shown as indicated on each image. **C**, Box plot showing the proportion of cells expressing the Confetti reporter (Confetti+), which costain for aSma and Mac3 (Confetti+ aSma+ Mac3+) within different plaque regions (7 plaques from 6 mice). Only red and blue plaques were used for this analysis as the 488 channel was required for aSma imaging. All data are from animals labeled at high density (10× 1 mg tamoxifen). CFP indicates cyan fluorescent protein.

Comparison of Confetti reporter expression with staining patterns for aSma and Mac3 within sequential cryosections revealed that individual clonal monochromatic regions often (27/37 regions in 23 plaques) contain both aSma- and Mac3-expressing cells. This suggests that VSMC-derived plaque cells displaying different phenotype were generated from a single VSMC, implying that individual VSMCs are highly plastic. To test this idea, we scored the position of 127 monochromatic regions in 82 plaques from 16 high density–labeled animals with respect to plaque cap and core (Online Table III). Some monochromatic regions were restricted to a specific sector of a lesion (11% for cap and 13% for core; Figure 4A) and often (>35%) >1 color was observed within either plaque core or cap (Online Figure IXA). However, most monochromatic regions (96/127) spanned both plaque domains (Figure 4A). Importantly, the frequency of such putative bipotent regions was significantly larger than expected by independent chance labeling of 2 proximate unipotent clones (χ^2^=228, *P*<0.01, 3 degrees of freedom, n=96; statistics analysis for each color in Online Data Supplement). In addition, supporting the conclusion that individual VSMCs can give rise to both fibrous cap and plaque core cells, monochromatic regions in animals with reduced labeling frequency also spanned both cap and core regions (14/17 regions from 14 plaques in 5 animals).

Costaining for aSma and Mac3 demonstrated a low, but consistent, proportion of lineage-labeled plaque cells that express markers of both phenotypes (Figure 4B). Double-positive cells were most abundant in the core and generally located in the region bordering the fibrous cap (Figure 4B and 4C). Interestingly, only 3 of >1100 cells scored costained for aSma and Mac3 without expressing the Confetti reporter, compared with 57 that were positive for aSma, Mac3, and the Confetti reporter, suggesting that coexpression of VSMC and macrophage markers is specific to VSMC-derived plaque cells. Recent single cell analysis similarly suggested coexpression of inflammatory and VSMC markers in VSMC-derived plaque cells, but cell-by-cell data were not presented precluding a direct comparison.^9^ Taken together, this analysis demonstrates that individual VSMCs have the ability to generate plaque cells of different phenotypes.

### Plasticity of VSMCs That Do Not Form Monochromatic Regions in the Plaque

**Figure 5.**
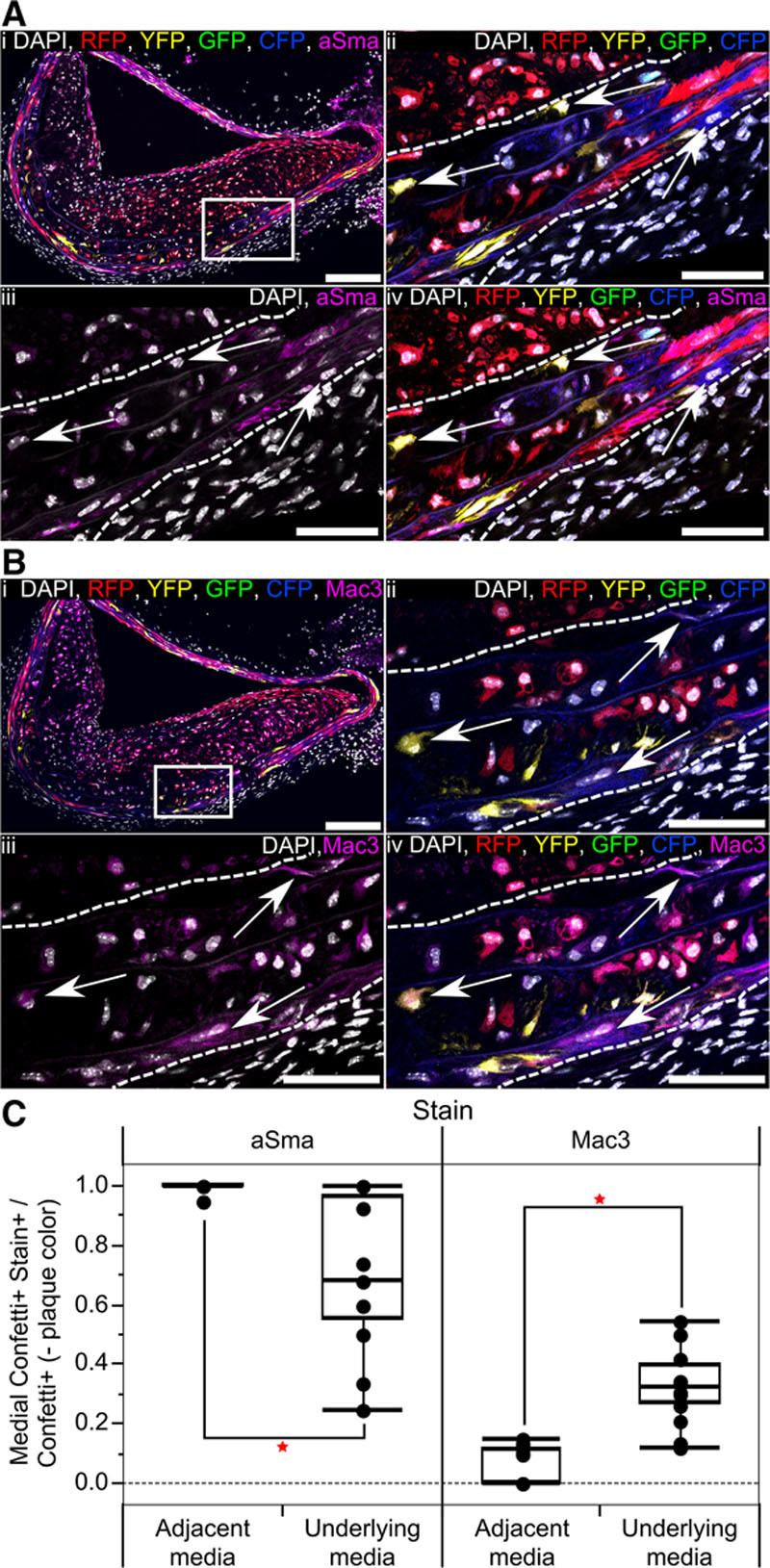
**Vascular smooth muscle cells (VSMCs) within the media directly underlying atherosclerotic plaques undergo phenotypic switching without contributing to the cell mass of the lesion. A** and **B**, Serial cryosections from artery of high density–labeled (10× 1 mg tamoxifen) animal containing a plaque with RFP+ VSMC–derived cells, stained for alpha smooth muscle actin (aSma) (**A**) and Mac3 (**B**). The region outlined in (i) is magnified in (ii through iv), white dotted lines outline the media. Signals for fluorescent proteins, nuclear DAPI (4',6-diamidino-2-phenylindole; white), aSma (magenta; **A**), and Mac3 (magenta; **B**) are shown as indicated on each image. Arrows in (**A**) point to aSma-negative cells within the media, which express the Confetti reporter (excluding RFP-expressing cells). Arrows in (**B**) point to Mac3-positive cells within the media, which express the Confetti reporter (excluding RFP-expressing cells). Scale bars are 150 μm in (i) and 50 μm in (ii through vi). **C**, Box plot quantifying the proportion of cells expressing the Confetti reporter, which stain positive for aSma or Mac3 (Confetti+ Stain+), relative to all cells expressing Confetti fluorescent proteins not found in the plaque (Confetti+, −plaque color) within the media underlying or adjacent to a plaque (based on 17 plaques from 6 animals). Red stars indicate a significant difference based on a 2-way analysis of variance, *P*<0.05. All data are from animals labeled at high density (10× 1 mg tamoxifen). CFP indicates cyan fluorescent protein; GFP, green fluorescent protein; RFP, red fluorescent protein; and YFP, yellow fluorescent protein.

In advanced lesions, the medial layer directly underlying plaques was often thickened with loss of the characteristic elongated cell and nucleus morphology of VSMCs (Figure 5A and 5B), indicating that phenotypic changes occur in cells that do not contribute to plaque development. To test this idea, we quantified marker expression in the media underlying plaques compared with the media of regions without visible plaque within the same tissue section. To ensure that the analysis was restricted to nonexpanding cells, we only analyzed medial cells expressing Confetti colors different from those present within the lesion. Relative to medial VSMCs adjacent to the plaque, which were nearly all aSma+ (median 100% and mean 99%), there was a significant reduction in aSma staining in the media under the plaque (25% to 100%, median 68%), suggestive of phenotypic switching of these cells (Figure 5C). This confirms previous reports.^6^ Correspondingly, expression of Mac3 was significantly more frequent in Confetti+ cells in the media under the plaque (median 32%) compared to adjacent media where Mac3-positive cells were rarely observed (median 0%; Figure 5C). This demonstrates that changes in VSMC phenotype occur independent of cell expansion but that these changes in medial cells are likely to be a response to the plaque environment and, therefore, a consequence rather than a cause of neointimal growth.

Interestingly, there was a modest, but not significant, reduction in the frequency of cells expressing the Confetti reporter within the media underlying the plaque compared with the adjacent media. Furthermore, whereas virtually no Mac3+ cells were observed in the adjacent media, a significant proportion of Confetti− Mac3+ cells were found in the media underlying plaques, suggesting an influx of Mac3-expressing cells (eg, from the bone marrow or adventitia), accompanying plaque development.

### VSMC Proliferation After Vessel Injury

**Figure 6.**
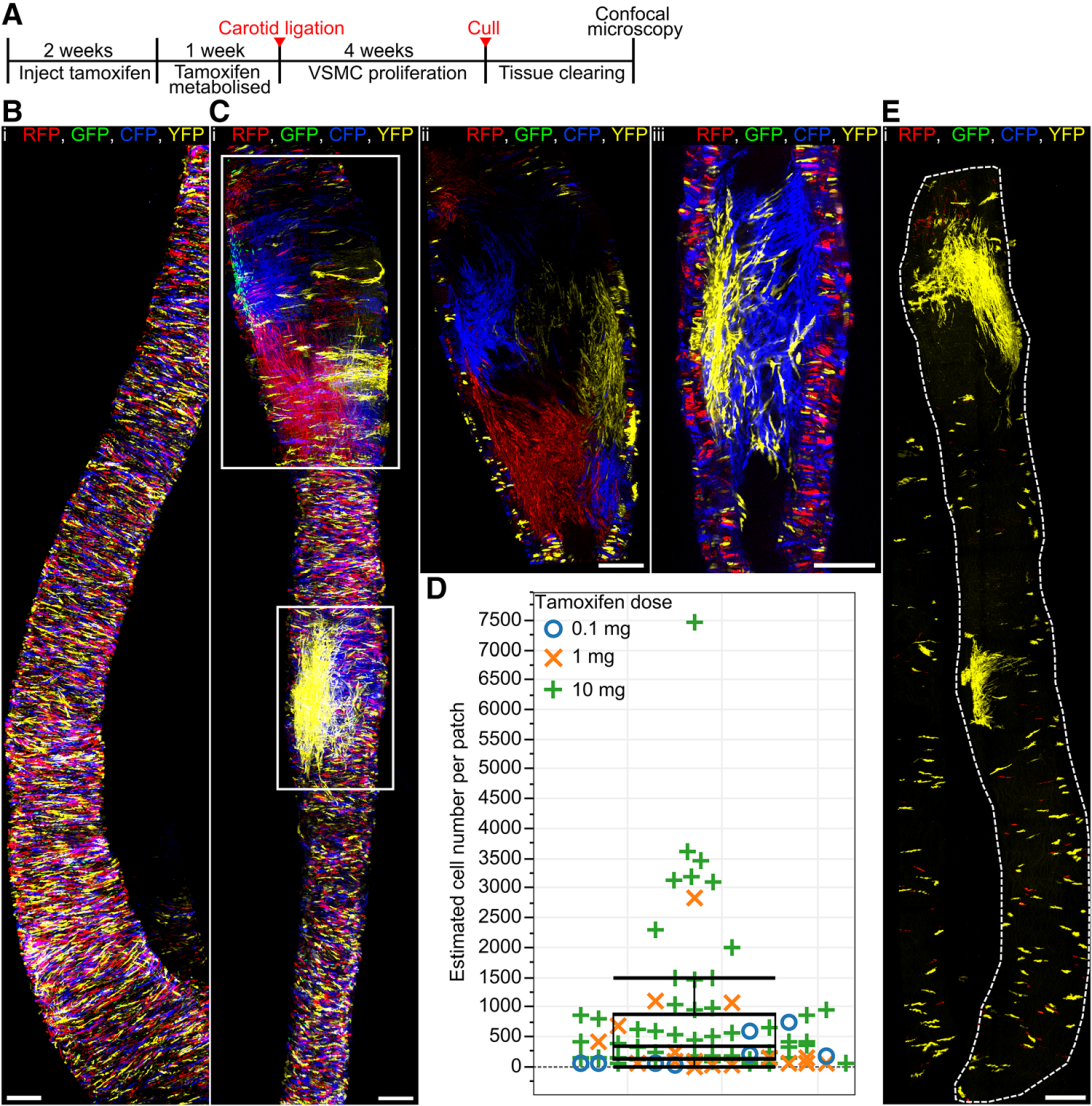
**A subset of vascular smooth muscle cells (VSMCs) proliferate to form the injury-induced neointima. A**, Experimental protocol for carotid artery ligation studies. **B** and **C**, Whole-mounted control right carotid artery (**B**) or ligated left carotid artery (**C**) from a high density–labeled animal (10× 1 mg tamoxifen) 28 days post surgery. Maximal projection of confocal Z-stack covering the entire artery is shown in (**B**) and (**C**[i]), whereas (**C**[ii] and **C**[iii]) show magnified longitudinal cross section of the regions outlined in (**C**[i]). **D**, Box plot showing the size of individual monochromatic patches. The patch sizes of low (blue circles) and medium density–labeled animals (orange crosses) largely fall within the interquartile range observed in high density–labeled animals (green crosses), strongly suggesting that patches result from clonal expansion of a single cell. Large patches (>1500 cells) might arise from merging of same colored clones. Fifty-two patches from 12 high density–labeled (10× 1 mg), 15 patches from 4 medium density–labeled (1× 1 mg), and 8 patches from 4 low density–labeled animals (1× 0.1 mg) were analyzed. **E**, Maximal confocal Z-stack projection of whole-mounted ligated left carotid artery from a low density–labeled animal (1× 0.1 mg tamoxifen), analyzed 28 days post ligation. Signals for fluorescent proteins are shown. All scale bars are 150 μm. CFP indicates cyan fluorescent protein; GFP, green fluorescent protein; RFP, red fluorescent protein; and YFP, yellow fluorescent protein.

To test whether the low frequency of proliferating VSMCs in advanced atherosclerotic lesions is a consequence of a low inflammatory signal from the lipid-rich diet or an inherent trait of the VSMC population, we labeled Myh11-CreERt2/Rosa26-Confetti animals at high frequency (10× 1 mg tamoxifen) before ligation of the left carotid artery (Figure 6A), which induces robust and rapid VSMC proliferation.^19^ As expected, confocal microscopy analysis of the whole-mounted carotid arteries revealed that VSMCs in the control right carotid artery did not proliferate (Figure 6B). In contrast, the neointima formed in the left carotid artery 28 days after surgery was composed of large contiguous monochromatic VSMC-derived patches, which occupied a defined volume. This observation suggests that the neointima is formed by clonal proliferation of a small proportion of VSMCs (Figure 6C). Intermingling of colors was only observed where ≥2 colored patches meet (Figure 6C[iii]). Most animals displayed 2 to 7 colored patches, but this ranged from 1 to 20 (Online Table IV) and correlated with the size of the remodeled area (*R*^2^=0.7). Similar to atherosclerotic plaques, patches of all colors were observed after ligation at frequencies comparable to the labeling frequency (Online Figure I), again confirming that recombination does not confer selective advantage to cells expressing a particular fluorescent protein.

Patch size ranged from 48 to >7000 with a median of 435 cells per patch (Figure 6D; Online Table IV). Importantly, the differences in patch size within the same vessel were on the same scale as interanimal variation, suggesting that this variation is not due to differences associated with the ligation surgery. To assess whether patches resulted from chance merging of independent clones of the same color, we generated low and medium density–labeled animals (≈1% and 40% recombination) using reduced tamoxifen doses (1× 0.1 and 1× 1 mg per animal). Remodeled arteries of animals with reduced labeling frequency presented isolated labeled patches comparable to those observed in high density–labeled animals (Figure 6D and 6E). However, median patch size was lower, suggesting that large clones (>1500 cells) could be the result of patch merging. These results demonstrate that a subset of Myh11-expressing cells undergoes multiple rounds of division to generate the neointima after vascular injury. On the basis of a VSMC density of ≈5000 cells/mm^2^,^20^ carotid artery diameter (470 μm),^21^ and on average 2.7±1.0 patches per mm remodeled artery (Online Table IV), we estimated that <0.1% of VSMCs contributed to the formation of the neointima 28 days post ligation. The finding that disease-associated VSMC accumulation results from clonal expansion of a restricted subset of VSMCs that express the mature SMMHC/Myh11 marker in 2 different animal models suggests that this is an inherent feature of this cell population.

### VSMC Plasticity After Vessel Injury

**Figure 7.**
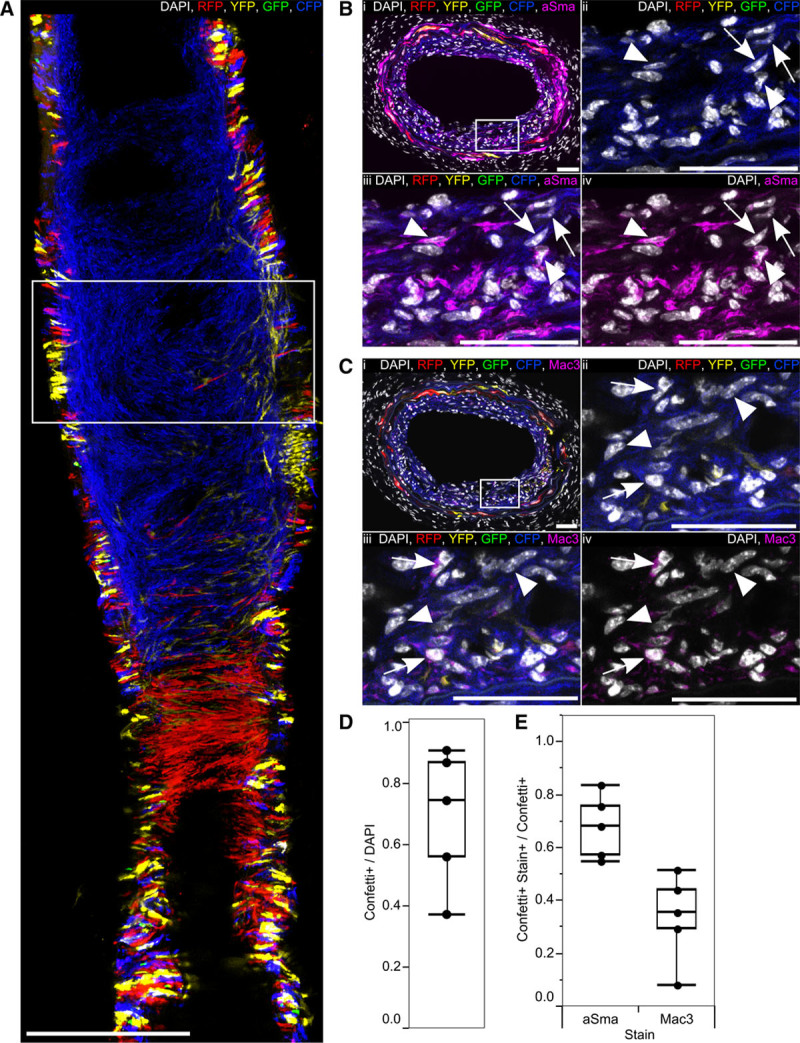
**Injury-induced vascular smooth muscle cell (VSMC)–derived neointima contains few phenotypically switched VSMCs. A**, Maximal projection of 3 central scans (7 μm apart) of a confocal Z-stack of whole-mounted ligated left carotid artery from high density–labeled (10× 1 mg tamoxifen) animal, 28 days post ligation. Scale bar is 300 μm. **B** and **C**, Transverse cryosections from the region outlined in (**A**), stained for alpha smooth muscle actin (aSma; **B**), or Mac3 (**C**); the region outlined in (i) is magnified in (ii through iv). Arrows point to cells expressing the Confetti reporter (Confetti+) that do not stain for aSma (**B**) and Confetti+ Mac3+ cells (**C**) within the neointima. Arrow heads point to Confetti+ aSma+ cells (**B**) or Confetti+ Mac3− cells (**C**) within the neointima. Scale bars are 50 μm. Signals for fluorescent proteins, nuclear DAPI (4',6-diamidino-2-phenylindole; white), aSma (magenta, **B**), and Mac3 (magenta, **C**) are shown as indicated on each image. **D**, Box plot showing the proportion of all cells (DAPI) within the neointima, which express the Confetti reporter (Confetti+). **E**, Box plot displaying the proportion of Confetti+ neointimal cells that stain positive for aSma or Mac3 (Stain+; based on 5 regions from 4 animals). All data are from high density–labeled (10× 1 mg tamoxifen) animals. CFP indicates cyan fluorescent protein; GFP, green fluorescent protein; RFP, red fluorescent protein; and YFP, yellow fluorescent protein.

To examine whether clonal proliferation after vascular injury was accompanied by similar phenotypic changes to those observed in atherosclerosis, we immunostained cross sections of arteries (after whole mount imaging) for aSma and Mac3 (Figure 7A through 7C). aSma was expressed in most VSMC-derived neointimal cells 28 days post surgery, although there was a reduction compared with healthy arteries (Online Figure X). In contrast to atherosclerotic plaques, there was little specific localization of aSma+ and aSma− cells expressing the Confetti reporter, although a degree of clustering of aSma-negative cells was observed in some samples (data not shown). We observed an upregulation of Mac3 in a subset of VSMC-derived cells (Figure 7C), but the expression level seemed to be lower than in Mac3+ Confetti+ cells within plaques (Figure 3B). These results demonstrate a clear difference in phenotypic switch–associated gene expression changes between the 2 disease models. We, therefore, tested whether VSMC-derived cells in injury-induced neointima show evidence of an earlier stage of phenotypic switching by staining for SMMHC, which marks fully mature VSMCs and has been reported to be downregulated early during phenotypic switching.^10^ However, the staining pattern for SMMHC was similar to aSma, suggesting that VSMCs in the neointima exist in a largely contractile state (Online Figure XIA).

### Migration of VSMCs in Disease

Interestingly, VSMC-derived neointimal patches were connected to patches expressing the identical Confetti color within the medial layer (Online Figure XII). This suggests that VSMC proliferation initiates in the media and that VSMC progeny then migrate through the elastic laminae, where they continue dividing to form the lesion (Online Figure XIB).^19^ Occasional medial patches without a linked neointimal patch of the same color were observed, indicating that proliferation is not strictly correlated with migration, but that these could be independent events. However, we did not find evidence for significant contribution of migration independent of proliferation to neointima growth, which would generate a mosaic neointima similar to the medial labeling pattern. In particular, coherent neointimal patches did not contain singlet VSMCs expressing other fluorescent proteins (Figure 6C), which would be observed if many cells from the randomly labeled medial wall migrate. This was substantiated by the absence of intermingling of cells expressing different Confetti colors, except at border regions between patches (Figure 6C[iii]).

Similarly, we did not find evidence that migration of nonproliferating cells contributes significantly to atherosclerotic plaque development. Most monochromatic regions formed a coherent structure and few (<10%) displayed intercalation of cells of different colors (for an example of intercalating plaque, Online Figure VIB). Monochromatic VSMC-derived plaque regions did contain interspersed nonlabeled cells (Figure 3A and 3B; arrows heads), which is consistent with the known infiltration of monocytes that contribute to lesion development. As VSMC labeling was not 100% efficient, we cannot rule out that these result from VSMC migration, but this is unlikely as we did not observe plaques that contained VSMC-derived cells expressing another Confetti color within a monochromatic region. We, therefore, suggest that migration of VSMCs independent of proliferation is a minor contribution to VSMC accumulation in vascular disease.

## Discussion

The multicolor lineage–tracing experiments presented here show that extensive proliferation of a small subset of mature, but plastic, VSMCs within major arteries generates neointimal VSMC-derived cells in 2 different vascular disease models. Proliferating VSMCs undergo multiple cell divisions to form large oligoclonal plaques, potentially explaining the replicative exhaustion, telomere shortening, and increased senescence observed in human plaques.^22,23^ Consistent with extensive proliferation of VSMC-derived cells, we observed a high proportion of EdU (5-ethynyl-2'-deoxyuridine)-positive Confetti-expressing cells within the neointima of animals that received repeated EdU injections after carotid ligation (Online Figure XIB). Our study correlates with and extends findings from LacZ-lineage tracing of SM22a-positive cells, which marks both mature and immature VSMCs.^7^ However, our data in normal adult mice contrast with findings that multiple existing cells proliferate to occlude the vessels of elastin-mutant embryos.^24^ This difference is most likely due to lineage labeling of less differentiated embryonic cells (E12.5) in that study^24^ compared with adult VSMCs here. The observation that a small fraction of Myh11-expressing VSMCs undergoes clonal expansion in 2 distinct disease models could suggest the existence of specialized cells within arterial walls. This is important as treatments that specifically target the proliferative VSMC population might retain the structural integrity of the artery after injury or in atherosclerosis.

Remarkably, most VSMC clones comprise both macrophage-like cells in the core and aSma-positive cap cells. This observation establishes that individual VSMCs show phenotypic plasticity as opposed to different VSMC subsets giving rise to different phenotypes. Interestingly, although advanced atherosclerotic plaques have low proliferative indices,^25^ EdU-labeled VSMC-derived cells were observed in both cap and core regions of established plaques, suggesting that cells displaying different phenotypes undergo cell division within the plaque (Online Figure XIII). Our observation that VSMC-derived cells expressing markers of both phenotypes are found at the border between the aSma-postive cap and the core region, containing macrophage-like VSMC-derived cells, (Figure 4B and 4C) opens the possibility that the VSMC-derived cells could interconvert between different phenotypic states depending on the environmental cues. This is important as it suggests that plaque stabilization could be achieved by therapeutic biasing of proliferating VSMCs toward the protective cap phenotype.

We did not observe differences in the frequency of proliferating VSMCs, their ability to contribute to both cap and core regions, or their bias toward aSma+ or Mac3+ phenotypes between plaques located in the descending aorta compared with the ascending aorta, aortic arch, and carotid arteries (Online Figure IX). This indicates a similar activation frequency and plasticity for VSMCs from different vascular regions, suggesting that there are no overt differences in plasticity of VSMCs generated from different embryonic origins (mesodermal for descending aortic VSMCs versus neural crest for VSMCs within aortic arch and carotid arteries^16^).

Our data suggest that VSMC proliferation and phenotypic switch–associated gene expression changes are not linked. For example, medial cells directly underlying the plaque downregulate aSma and express Mac3 without contributing to plaque mass, and clonally expanded VSMC-derived cells in the injury-induced neointima maintain aSma expression. Furthermore, we show that migration of VSMCs does not make a major contribution to VSMC accumulation in vascular diseases. Our study, therefore, adds in vivo single cell evidence supporting a notion that different aspects of VSMC phenotypic switching, including VSMC migration, contractile marker expression, and proliferation, could be regulated independently.

It is not clear why only a small proportion of VSMCs contributed to the observed disease-associated VSMC accumulation, in light of the general response of VSMCs reported after vessel injury.^26^ Interestingly, studies using radiolabeled thymidine to measure cell division in pig and rat models of vascular disease suggested that cell proliferation occurs more generally in the media.^27,28^ Those studies analyzed animals at earlier time points of disease, as opposed to our analyses of later stages. The apparent difference in the fraction of proliferating medial cells found in those studies and the restricted number of VSMCs we observe contributing to the neointima might, therefore, be due to the differences in time of analysis. For example, clonal selection or cell competition could occur at later time points after a more general response. Alternatively, the different phenotypes could arise from differences between experimental models or the inability to discriminate between VSMCs and infiltrating cells in the previous studies, which did not use lineage labels.^27,28^ In any case, it will be interesting to study why the observed phenotypic changes occurring generally in all VSMCs is reversible in most cells but not in a few of the cells. The restriction of migration through the elastic laminae to expanding cells might suggest that the expression of matrix-remodeling factors could be involved. One possibility is that the differential activation of VSMCs we observe is due to the observed heterogeneity between cells in healthy vessels.^10^ Future studies should reveal whether limitation of VSMC contribution to disease-associated cell accumulation in major arteries to a few mature VSMCs that proliferate extensively is associated with somatic mutation,^29^ a preselected progenitor pool primed by epigenetic heterogeneity, as suggested for neomuscularization of distal pulmonary arterioles,^30^ or due to the rare stochastic activation of an equipotent population.

## Acknowledgments

We thank Jenny Nichols, Doug Winton, Filipe Lourenço, and Crystal McClain for materials and advice; Petko N. Petkov for help with data analysis; Veronique Azuara for comments on the article; and Alison Finigan, Nichola Figg, and Lauren Baker for technical assistance.

## Sources of Funding

This work was funded by the British Heart Foundation grants to H.F. Jørgensen (PG/12/86/29930 and FS/15/38/31516). H.F. Jørgensen and M.R. Bennett acknowledge support from the British Heart Foundation (BHF) Oxbridge Centre of Regenerative Medicine (RM/13/3/30159) and the BHF Cambridge Centre of Research Excellence (RE/13/6/30180). B.D. Simons acknowledges the support of The Wellcome Trust (098357/Z/12/Z). Light microscopy core facilities at Cancer Research UK Cambridge Research Institute, Cambridge Advanced Imaging Centre, and the Wellcome Trust-Medical Research Council, Institute of Metabolic Science, Metabolic Research Laboratories, Imaging core, Wellcome Trust Strategic Award [100574/Z/12/Z] are acknowledged for microscopy and the Cambridge National Institute for Health Research Biomedical Research Centre Cell Phenotyping Hub for flow cytometry. The other authors report no conflicts.

## Disclosures

None.

## Supplementary Material

## Novelty and Significance

### What Is Known?

- Vascular smooth muscle cell (VSMC) accumulation is a hallmark of vascular disease, including atherosclerosis.
- Mature VSMCs retain phenotypic plasticity, and VSMC-derived cells within atherosclerotic plaques can adopt, among others, a macrophage-like state.
