# Supplementary material for: Extensive Proliferation of a Subset of Differentiated, yet Plastic, Medial Vascular Smooth Muscle Cells Contributes to Neointimal Formation in Mouse Injury and Atherosclerosis Models
Source: Circ Res. 2016 Sep 28;119(12):1313–23. doi: 10.1161/CIRCRESAHA.116.309799 (PMC5149073; doi:10.1161/CIRCRESAHA.116.309799)
Supplement: Supplementary file 1 [file res-119-1313-s001.pdf]

SUPPLEMENTAL MATERIAL

Supplemental Methods

Supplemental References

Supplemental Legends (Online Figures I-XIII, Online Video I)

Online Tables (I-IV)

Online Figures (I-XIII)

Online Video I (separate file)

Supplemental Methods

Animal Experiments

All animal experiments have been approved by the UK Home Office (PPL70/7565), were performed according to Home Office guidelines and were approved by the local ethics committee. The Myh11-CreERt2[1], Rosa26-Confetti[2], ApoE-/-[3] mouse lines have all been described. Myh11-CreERt2 and ApoE$^{-/-}$ were on an inbred C57Bl/6 background (for at least 10 generations), whereas the Rosa26-Confetti allele was on a mixed C57Bl6/BALB/c background prior to crossbreeding. We used experimental animals that had been crossbred between one and six generations (see Online Table II), with no observed difference in the described phenotype. The Myh11-CreERt2 transgene is Y-linked, so all experimental animals in this study were males. Recombination was induced in 6-8 week old animals by intraperitoneal injections of 10x 1 mg (high density-labeling), 1x 1 mg (medium density-labeling) or 1x 0.1 mg (low density-labeling) tamoxifen (Sigma) in corn oil followed by a rest period for at least 1 week before inducing VSMC proliferation by either high fat feeding or carotid ligation surgery. For carotid ligation experiments, Myh11-CreERt2+, Rosa26-Confetti+ animals were anaesthetized with 2.5-3% isofluorane (by inhalation) and given pre-operative analgesic (Temgesic, Buprenorphine). For carotid artery ligation, the left carotid artery was tied just below the bifurcation point. Both the right and left carotid arteries were removed 28 days post-surgery and processed for microscopy. For the atherosclerosis model, Myh11-CreERt2+, Rosa26-Confetti+, ApoE-/- animals were given a high fat diet (Western Rd (p) Product code:829100 SDS, containing 21% fat and 0.2% cholesterol) for 16-19 weeks (except where animals had to be culled prematurely, see Online Table II) starting 1 week after the final tamoxifen injection after which the aorta and carotid arteries were removed.

Tissue Processing

Mice were culled by CO_2 asphyxiation and arteries were perfused with PBS. The isolated arteries were dissected free from adipose and connective tissue, fixed (20 minutes at room temperature in 4% (v/w) paraformaldehyde in PBS), washed in PBS, stained with 4',6-diamidino-2-phenylindole (DAPI, 1 µg/ml in PBS, 1h at room temperature) and cleared overnight at 4˚C in RapiClear 1.52 (Sunjin lab). Carotid arteries from post-surgery animals were whole mounted in RapiClear 1.52 prior to whole mount confocal microscopy. Post-imaging, carotid arteries were cut transversely into 14 µm thick sections (cryosectioning after overnight equilibration in 30% sucrose/PBS and embedding in TissueTek O.C.T.). The plaque-filled arteries from animals on high fat diet cleared less efficiently and were therefore immediately cut transversely into 50-100 µm (vibratome sectioning after embedding in 4% low melt agarose/ PBS) or 20 µm sections (cryosectioning after overnight equilibration in 30% sucrose/PBS and embedding

in TissueTek O.C.T.). To assess labeling specificity, non-arterial tissue (skeletal muscle, liver, lung) were fixed (20 minutes at room temperature in 4% (v/w) paraformaldehyde in PBS), embedded in 4% low melt agarose and sectioned (100 μm) using a vibratome. Sections were mounted in RapiClear 1.52 and imaged by confocal microscopy.

Immunostaining

Serial cryosections were stained for markers of VSMC-derived cell populations or subjected to Haematoxylin/Eosin staining. For immunostaining, sections were briefly rinsed in PBS, permeabilized in 0.3% Triton X-100 in PBS (20 min at room temperature) and incubated for 1h at room temperature in blocking buffer (1% bovine serum albumin, 10% normal goat serum in PBS). Staining with the following primary or isotype control antibodies diluted in blocking buffer was done overnight at 4˚C (aSMA - biotin, Abcam, 2.5 μg/ml, ab125057; aSMA - Alexa Fluor 488, Abcam, 2.5 μg/ml, ab184675; Mac3 - Alexa Fluor 647, Biolegend, 2.5 μg/ml, 08511; Myh11, Abcam, 2.5 μg/ml, ab53219; Mouse IgG2a, k - biotin, Biolegend, 2.5 μg/ml, 400203; Rat IgG1, k - Alexa Fluor 647, Biolegend, 2.5 μg/ml, 400418; Rat IgG2a, k - Alexa Fluor 647, Biolegend, 2.5 μg/ml, 400526; Rabbit IgG, Abcam, 2.5 μg/ml, ab27478). Sections were then washed 3 times in PBS and incubated with a secondary antibody where necessary (Streptavidin - Alexa Fluor 647, Biolegend, 0.5 μg/ml, 405237; Goat Anti-Rabbit IgG - Alexa Fluor 647 0.5 μg/ml, ab150079). Nuclei were stained with DAPI, (1 μg/ml in PBS, 10 min at room temperature) and mounted in RapiClear 1.52.

Edu administration and detection in carotid ligation and atherosclerosis experiments

For carotid ligation experiments EdU was dissolved in PBS (5 mg/ml) and injected intraperitoneally (200 μl EdU per injection) starting the day after carotid ligation and every day Monday to Friday following this until they were culled, totalling 19 (200 μl) injections over 28 days. For atherosclerosis experiments EdU (5 mg/ml) was injected intraperitoneally (300 μl) three hours before mice were culled at the end of their 16 weeks of high fat diet. Detection was performed using the Click-iT® Plus Edu Alexa Fluor® 657 Imaging kit (Life Tech, C10640) on cryosections according to the manufacturer's instructions.

Imaging and image processing

Imaging was done using confocal laser scanning microscopy (Leica SP5 or SP8) with laser lines and detectors set for maximal sensitivity without spectral overlap for DAPI (405 laser, 417-508 nm), CFP (458 laser, 454-502 nm), GFP (488 laser, 498-506 nm), YFP (514 laser, 525-560 nm), RFP (561 laser, 565-650 nm) and Alexa Fluor 647 (633 laser, 650-700 nm).

For whole mount samples, a 20x oil objective was used and tile scans of Z-stacks totalling ~400 μm (~6-8 μm between each Z-section) acquired. Cryosections were imaged using a 40x oil objective, tiled Z-stacks typically totalled 20 μm (2- 4 μm separation per Z-section). Vibratome sections were imaged using a 20x oil objective with a Z-stack typically totalling 100 μm (~5 μm between each Z-section). Data was acquired at an optical section resolution of 1024 x 1024 and tiles were stitched using LAS software (Leica).

Imaris 8 software was used for image processing and analysis of whole mount and vibratome samples. Processing includes brightness and contrast adjustments, generation of maximal projections, virtual cross sectioning, animation and surface rendering. For calculation of patch size within whole mount samples, we modelled the surface of individual patches using the surface rendering function within Imaris. A 'patch' was defined as a large contiguous mass of cells which share the same color and occupy both neointimal and medial compartments of the vessel. To estimate cell number per patch, the surface volume was divided by the volume of an average of 8 individual VSMCs of the same color.

Images are maximal Z-projections (generated in Imaris) where indicated in figure legends or individual scans from a confocal Z-stack (generated in FIJI).

Plaque and neointima scoring

Plaques were analyzed from three regions within the vasculature; the carotid arteries (CA) comprising the region from where the right and left CA bifurcate to where they meet the aorta; the aortic arch (Arch) comprising the ascending aorta proximal to the aortic root and the arch region; and the descending aorta (DA) comprising the region distal to the Arch region to the bifurcation of the abdominal aorta.

For quantification of imaged cross sections, plaques were separated into three distinct regions; the cap region, which was defined as the organized layer of elongated cells on the inner most surface of the plaque adjacent to the lumen; the core region, which was defined as a disorganized mass of cells towards the centre of a plaque; and the shoulder region, which was defined as the mass of cells within the plaque which are immediately adjacent to the arterial wall on either side of the plaque.

The neointima within carotid ligation samples was defined as the area between the inner elastic lamina (IEL) of the artery wall and the lumen.

Quantification of labeled (Confetti+) and marker positive (Stain+) cells was performed on immunostained cryosections (20 μm for plaque and 14 μm for ligated arteries). Cells within an area of 1 mm^2 were counted for each plaque region and neointima (described above). For almost all plaque regions/neotintima (>60%, except where they were too small) two areas were scored for each and the average frequency used. Cell scoring was performed on confocal Z-stacks in Imaris in 3D to evaluate staining within Z-sections below and above the cell of interest. This ensures that counts are made on a cell by cell basis in the high resolution Z-stacks.

T-test and two-way ANOVA test were performed within R (reference[5]) on selected variables to determine significant differences ($p<0.05$) as indicated in figures.

Calculating theoretical distribution of plaque colors

To compare the observed distribution of colors per plaque to what would be expected if plaques were generated from proliferation of 1, 2, 3 or 4 cells, we calculated the theoretical distribution as follows. All possible combinations of colored cells were considered (e.g. if 3 cells proliferate there are 64 possible color combinations: red-red-red, red-red-green, red-green-red, red-yellow-blue…). To calculate the probability of each color combination, we applied the known recombination frequency of each color (for example: red-red-red = 0.34 x 0.34 x 0.34, red-red-green = 0.34 x 0.34 x 0.07, red-green-red = 0.34 x 0.07 x 0.34, red-yellow-blue = 0.34 x 0.25x 0.33…). The frequencies for color combinations of 1, 2, 3 and 4 colors were summed; for example, out of the 64 possible combinations of 3 cells there are 4 yielding one color (red-red-red etc.) with a summed frequency of 0.063, 36x two color combination (summed frequency=0.563), 24x three color combinations (summed frequency=0.375) and 0 x four color combinations. This data is shown in Figure 2G for comparison to the observed distribution shown in Figure 2F.

Statistics analysis of bipotency

Chi^2 testing was used to assess whether monochromatic regions occupying both cap and core were observed more frequently that what would be expected by chance.

To address the question of bipotency for each Confetti color, we want to compare the observed data against the null hypothesis that “the VSMC cap and core progenitors are unipotent”. The fact that we see some plaques with lineage labeled cells in either the cap or the core but not both suggests that, following activation, at least some of the cells are unipotent. We wish to assess whether they all could be.

To this end, we first assume that contiguously labeled patches in the cap or the core region derive from clonal events. Then, according to the null hypothesis, the probability that a patch in the core will be labeled in color c is given by

$$P_{\text{core},c} = f q_c$$

where f denotes the fraction of fluorescently labelled cells, estimated at around 0.8 in the densely labeled sample, and $\mathrm{q_c}$ denotes the relative labeling efficiency of color c. Similarly, the probability that a patch in the cap is labeled in color c is given by $\mathrm{P_{cap,c} = P_{core,c}}$. Then, the chance that we have a clone of color c in the core but not the cap is given by

$$P_{\text{core},c}(1 - P_{\text{cap},c})$$

(similarly cap but no core), while the probability that both core and cap acquire color c is given by

$$P_{\text{core},c} P_{\text{cap},c}$$

If we then focus on the ensemble of clones that contain color c in the cap, the core or both, the relative probabilities must be normalized by the factor

$$P = P_{\text{core},c}\left(1 - P_{\text{cap},c}\right) + P_{\text{cap},c}\left(1 - P_{\text{core},c}\right) + P_{\text{core},c} P_{\text{cap},c}$$

so that the relative chance of finding a plaque with core labeling of color c

$$r_{\text{core},c} = \frac{P_{\text{core},c}\left(1 - P_{\text{cap},c}\right)}{P}$$

which is equal to $r_{\text{cap},c}$, while the relative chance of finding both labeled in color c is given by

$$r_{\text{both},c} = P_{\text{core},c} P_{\text{cap},c} / P$$

Finally, for an ensemble of clonal events, we expect that the statistical error on the measured fractions should become defined by the standard error of the mean, given by

$$\left(r_{\text{core},c}(1 - r_{\text{core},c})/N\right)^{1/2}$$

where N denotes the total number of plaques sampled that contain color c.

Thus, the observed Cap+core frequencies are significantly higher for all four Confetti colors:

RFP	Cap	Core	Cap+core	N
Observed	6	9	48	63
Calculated (r*N)	26	26	11	
error (SEM)	1.6	1.6	0.5	

YFP	Cap	Core	Cap+core	N
Observed	3	4	20	27
Calculated (r*N)	12	12	3	
Error (SEM)	1.1	1.1	0.2	

nGFP	Cap	Core	Cap+core	N
Observed	1	1	8	10
Calculated (r*N)	5	5	0.4	
Error (SEM)	0.8	0.8	0.02	

mCFP	Cap	Core	Cap+core	N
Observed	4	3	20	27
Calculated (r*N)	12	12	4	
Error (SEM)	1.1	1.1	0.2	

"Observed" is the measured number of cap only, core only and cap+core clones, "Calculated" is the expected number against the null hypothesis, and "Error" denotes the expected error (SEM) given the total number of clones that are measured (N).

Flow cytometry
Single cell suspensions of VSMCs were obtained by enzymatic digestion of mouse aortas essentially as described previously[4]. Briefly, following removal of adipose and connective tissue, the samples were pre-digested in an enzyme mix (1 mg/ml Collagenase, Gibco, and 1 U/ml Elastase, Worthington, in Dulbecco's Modified Eagles Medium) for 10 min at 37 ˚C after which the adventitia was peeled off and endothelial cells removed by gentle scraping. The medial layer was then incubated in fresh enzyme mix for 1-2 h with gentle agitation. Bone marrow was isolated from the femur and tibia and cells passed through a 40 µm cell strainer. Blood was collected by cardiac puncture following CO_2 asphyxiation and red blood cells lyzed by incubation in 55 mM NH4Cl, 10 mM KHCO3, 0.1 mM EDTA (pH 7.3) for 10 min. Cell suspensions were then washed 3 times in PBS and analyzed on a BD Fortessa flow cytometer. Gates for fluorescent protein detection were defined based on VSMCs from wild type and labeled confetti animals.

Supplemental references

1. Wirth A, Benyó Z, Lukasova M, Leutgeb B, Wettschureck N, Gorbey S, Örsy P, Horváth B, Maser-Gluth C, Greiner E, Lemmer B, Schütz G, Gutkind JS, Offermanns S. G12G13-LARG-mediated signaling in vascular smooth muscle is required for salt-induced hypertension. Nature medicine. 2008;14:64–68.
2. Snippert HJ, van der Flier LG, Sato T, van Es JH, van den Born M, Kroon-Veenboer C, Barker N, Klein AM, van Rheenen J, Simons BD, Clevers H. Intestinal crypt homeostasis results from neutral competition between symmetrically dividing Lgr5 stem cells. CELL. 2010;143:134–144.

3. Piedrahita JA, Zhang SH, Hagaman JR, Oliver PM, Maeda N. Generation of mice carrying a mutant apolipoprotein E gene inactivated by gene targeting in embryonic stem cells. Proceedings of the National Academy of Sciences. 1992;89:4471–4475.

4. Hamblin M, Chang L, Chen YE. Isolation and Culture of Vascular smooth Muscle Cells. Oxford, UK: John Wiley & Sons, Ltd; 2013:125–130.

5. R Core Team (2016). R: A language and environment for statistical computing. R Foundation for Statistical Computing, Vienna, Austria. URL https://www.R-project.org/.

Legends to Online Figures and Video

Online Figure I: Frequency of Confetti reporter colors
Bar chart showing the proportions of each of the Confetti colors in VSMCs directly after recombination (light gray), in monochromatic regions within atherosclerotic plaques (dark gray) and in carotid ligation-induced neointimal patches (black). All data is from high density-labeled animals (10x 1 mg tamoxifen).

Online Figure II: Mosaic labeling of medial VSMCs
A, Longitudinal cross section of a whole mount carotid artery from a Confetti animal labeled at high density (10x 1 mg tamoxifen). Signals for fluorescent proteins are shown with (**i**) and without (**ii**) nuclear DAPI (white). Arrows point to labeled VSMCs within the medial layer, arrow heads point to unlabeled cells within the endothelium and adventitia. The luminal side is denoted as **"L"** and the adventitial side marked **"A"**. Scale bar is 50 µm.

Online Figure III: Specific labeling of VSMCs in Rosa26-Confetti+, Myh11-CreERt2+ animals
A, Flow cytometry analysis demonstrating background levels of fluorescence in bone marrow and peripheral blood from non-tamoxifen treated wild type animals and experimental Rosa26-Confetti+, Myh11-CreERt2+ animals labeled at high density (10x 1 mg tamoxifen). Samples were gated for live cells (FSC-A, SSC-A) and singlets (FSC-A, FSC-H). Negative cells were identified based on gates set on VSMCs from wild type and Confetti animals. **B,** Confocal microscopy images showing absence of recombination in liver (**i, iv**), skeletal muscle (**ii, v**) and lung (**iii, vi**) in Confetti animals after tamoxifen treatment (10x 1 mg). Note, that labeled cells in (**vi**) correspond to VSMCs lining a blood vessel. Signals for fluorescent proteins are shown with (**i, ii, iii**) and without nuclear DAPI staining (white, **iv, v, vi**). Scale bars are 100 µm.

Online Figure IV: Histological analysis of monochromatic plaque region
A, Haematoxylin and eosin staining of 20 µm cryosections from HFD treated Confetti animal labeled at high density (10x 1 mg tamoxifen). For comparison, the signals from fluorescent proteins in an adjacent section are shown with (**B**) and without nuclear DAPI staining (white, **C**). Scale bars are 100 µm.

Online Figure V: Proportion of plaque cells expressing the Confetti reporter
A, Box plot quantifying the proportion of all cells (DAPI) within plaques from different regions of the vasculature which express the Confetti reporter (Confetti+). **B, C,** Box plots showing the proportion of cells expressing the Confetti reporter (Confetti+), which were stained for either aSma or Mac3 (Confetti+ Stain+) within plaques from different regions of the vasculature (**B**) or different animals (**C**). Red stars indicate a significant difference based on a two-way ANOVA, $p<0.05$, data from 23 plaques from 6 animals labeled at high density (10x 1 mg tamoxifen). Arch: aortic arch, DA: descending aorta, CA: carotid artery.

Online Figure VI: Clonal VSMC expansion in atherosclerotic plaques
Examples of monochromatic plaques (**A**) and plaques containing VSMC-derived cells of more than one color (**B-D**). **B,** example of a plaque containing two intermingled clones (yellow and green). The section shown in panel (**D**) is cut through the top of the aortic arch and includes a portion of the right carotid artery. Signals from fluorescent proteins in 50-100 µm vibratome sections are shown with (**i**) and without nuclear DAPI staining (white, **ii**). All plaques are from animals labeled at high density (10x 1 mg tamoxifen). Scale bars are 100 (**A-C**) and 200 µm (**D**). A video of the plaque shown in panel **A** is provided as Supplemental Online Video I.

Online Figure VII: Animals labeled at medium density develop plaques with large monochromatic regions, which span both the cap and core
A, An atherosclerotic plaque from a medium density-labeled (1x 1 mg tamoxifen) Confetti animal following 16 weeks of high fat diet. Signals from fluorescent proteins are shown with (**i**) and without (**ii**) nuclear DAPI (white). Scale bars are 100 µm.

Online Figure VIII: Proportion of all cells within atheroscleotic plaques and injury-induced neointima which are VSMC-derived cells and express phenotype marker proteins
A-C, Box plots displaying the proportion of all plaque cells (DAPI) co-expressing the Confetti reporter and either aSma or Mac3 (Confetti+ Stain+) according to plaque region (**A**), individual animals (**B**) and vascular region (**C**) in high density-labeled (10x 1 mg tamoxifen) Rosa26-Confetti+, Myh11-CreERt2+, ApoE-/- animals after high fat diet. **D,** Proportion of total number of cells (DAPI) which co-express the Confetti reporter and either aSma or Mac3 (Confetti+ Stain+) in carotid ligation-induced neointima from high density-labeled (10x 1 mg tamoxifen) Rosa26-Confetti+, Myh11-CreERt2+ animals 28 days post-surgery.

Online Figure IX: Number and distribution of Confetti colors within atherosclerotic plaques
A-E, Bar charts showing the proportion of plaques in high density (10x 1 mg tamoxifen, **A, C-E**) and medium density (1x 1 mg tamoxifen, **B**) labeled animals with one, two, three or four colors. Bars represent the entire plaque (dark gray), the cap (light gray) or the core of the plaque (black). **C, D, E,** Data is shown for plaques in different vascular regions (**C,** Aortic Arch; **D,** Descending aorta; **E,** Carotid artery). **F,** Bar chart showing the proportion of monochromatic regions within plaques which occupy the plaque cap only (dark gray), core only (light gray) or both the core and cap (black). Data is shown for high density (10x 1 mg), low density-labeled (1x 1 mg) or all animals at the left, and stratified according to vascular regions on the right. Arch: aortic arch, DA: descending aorta, CA: carotid artery.

Online Figure X: Right carotid artery immunostaining controls
A-D, Single confocal scans from Z-stacks of 14 µm cryosections from the control right carotid artery of high density-labeled (10x 1 mg tamoxifen) animal 28 days after ligation of the left carotid artery stained for aSma (**A**), Mac-3 (**B**), Smmhc/Myh11 (**C**) or EdU (**D**). For each staining, (**ii-iv**) are zoomed images of the regions outlined in (**i**). Signals for fluorescent proteins, DAPI (white), aSma (magenta), Mac3 (magenta), EdU (magenta), and Smmhc (magenta) are shown as indicated on each image. Scale bars are 50 µm.

Online Figure XI: Smmhc and EdU staining in carotid ligation-induced neointima
A, B, Single confocal scans from Z-stacks of 14 µm cryosections from the left carotid artery 28 days post-ligation in a high density-labeled animal (10x 1 mg tamoxifen) stained for Smmhc (**A**) or EdU (**B**). For each staining, the region outlined in (**i**) is magnified in (**ii-iv**). Arrows point to cells co-expressing the Confetti reporter and Smmhc (**A**) or EdU (**B**), whereas arrow heads point to cells that express the Confetti reporter, but do not stain for the

respective marker. Signals for fluorescent proteins, DAPI (white), Smmhc (magenta) and EdU (magenta) are shown as indicated on each image. Scale bars are 50 µm.

Online Figure XII: Clonal patches span both media and neointima
Longitudinal cross section of the left carotid artery from animals labeled at high density (10x 1 mg tamoxifen, **i**) or low density (1x 0.1 mg tamoxifen, **ii**), which were analyzed 28 days post-surgery, showing that individual VSMC-derived clones are "anchored" in the medial layer. A single scan of a confocal Z-stack, which intersects the middle of the vessel, is shown. Signals for fluorescent proteins and DAPI (white) are shown. Scale bars are 100 µm.

Online Figure XIII: Proliferative VSMC-derived cells detected within both the core and cap of atherosclerotic plaques
A, B, Cryosections of atherosclerotic plaques from Confetti animals subjected to 16 weeks of high fat diet and injected with EdU 2 hours before analysis. The regions outlined in (**i**) are magnified in (**ii-iv**). Arrows point to EdU-positive cells expressing the Confetti reporter in the cap (**A**) or core region (**B**). Signals for fluorescent proteins, DAPI (white) and EdU (magenta) are shown as indicated on each image. **C, D,** Box plot showing the proportion of cells expressing the Confetti reporter (Confetti+) which are EdU+ (Confetti+ EdU+) displayed for all areas analyzed (**C**) and separately for the plaque cap, core and shoulder regions (**D**). **E,** Box plot showing the proportion of all cells (DAPI) which are EdU+. Data in (**C-E**) is from 9 plaques from 5 animals. All data is from high density-labeled animals.

Online Video I: Clonal VSMC expansion in atherosclerotic plaques
This video shows a 100 µm thick vibratome section from a carotid artery containing an atherosclerotic plaque formed of VSMCs of one color. Initially only GFP signal is shown, then YFP, CFP and RFP signals are added and finally the signal from nuclear DAPI staining (white). During the video DAPI is removed and shown again while the video demonstrates that VSMCs of a single color can occupy core and cap regions in a thick slice from an atherosclerotic plaque. The plaque shown in the video is also shown (Z-stack projection) in Online Figure VI, A. The scale bar and displayed size adjusts automatically as the video is played.

Online Table I. Efficiency of VSMC labeling in high density (10x 1 mg tamoxifen) and low density-labeled animals (1x 0.1 mg tamoxifen)

Cell I.D., Tamoxifen dose	Fluorescently labeled (%)			Non-labeled (%)		
	Average	Max	Min	Average	Max	Min
**VSMC wild type, 10 mg (n=1)**	0	NA*	NA*	100	NA*	NA*
**VSMC Confetti, 10 mg (n=6)**	78.5	92.9	69.6	21.5	30.4	7.1
**VSMC Confetti, 0.1 mg (n=3)**	0.8	1.0	0.5	99.2	99.5	99.0

*NA: Not applicable

Online Table II: Experimental animals used for atherosclerosis studies

Animal no.	Back-crosses*	Tamoxifen dose (mg)	HFD (weeks)	Plaques analyzed	Analysis method
1	2	10	17	4	vibratome
2	2	10	12†	2	vibratome
3	2	10	19	10	vibratome
4	2	10	18	8	cryo
5	2	10	18	8	cryo
6	2	10	18	7	cryo
7	2	10	14†	9	cryo
8	2	10	18	8	cryo
9	2	10	17	10	cryo
10	2	10	17	1	vibratome
11	2	10	16	2	cryo
12	2	10	16	2	cryo
13	2	10	16	4	cryo
14	2	10	16	3	cryo
15	2	10	16	3	cryo
16	2	10	16	1	cryo
17	2	1	16	2	cryo
18	2	1	16	3	cryo
19	2	1	16	4	cryo
20	2	1	16	4	cryo
21	2	1	16	1	cryo
22	2	0.1	13†	0‡	vibratome
23	2	0.1	11†	0	vibratome
24	2	0.1	14	0	vibratome
25	2	0.1	14	0	vibratome

* Number of backcrosses to C57Bl/6.

† Animals were analyzed prematurely due to health issues unrelated to cardiovascular defects (e.g. fighting or warts).

‡ One monochromatic region was observed, but this was not included in any analysis.

Online Table III: Plaque information

Animal no.	Plaque no.	Tamoxifen dose (mg)	Vascular Region*	Colors per plaque	aSma	Mac3	DS†	EdU
1	1‡	10	CA	2 (RFP, YFP)	-	-	-	-
1	2	10	DA	2 (RFP, YFP)	-	-	-	-
1	3	10	AA	1 (RFP)	-	-	-	-
1	4	10	CA	2 (YFP, nGFP)	-	-	-	-
2	5	10	AA	2 (RFP, mCFP)	-	-	-	-
2	6	10	AA	2 (nGFP, RFP)	-	-	-	-
3	7	10	CA	2 (nGFP, RFP)	-	-	-	-
3	8‡	10	CA	2 (YFP, nGFP)	-	-	-	-
3	9‡	10	AA	2 (RFP, mCFP)	-	-	-	-
3	10	10	AA	2 (RFP, YFP)	-	-	-	-
3	11‡	10	AA	2 (RFP, mCFP)	-	-	-	-
3	12	10	CA	1 (RFP)	-	-	-	-
3	13	10	CA	1 (RFP)	-	-	-	-
3	14	10	CA	2 (RFP, YFP)	-	-	-	-
3	15	10	CA	1 (YFP)	-	-	-	-
3	16	10	AA	2 (RFP, nGFP)	-	-	-	-
4	17	10	CA	1 (YFP)	-	-	-	-
4	18	10	CA	2 (mCFP, nGFP)	+	+	-	-
4	19	10	AA	2 (RFP, mCFP)	-	-	-	-
4	20	10	AA	2 (RFP, YFP)	-	-	-	-
4	21	10	AA	1 (RFP)	+	+	+	-
4	22	10	AA	1 (YFP)	-	-	-	-
4	23‡	10	DA	1 (RFP)	+	+	+	-
4	24	10	?	1 (RFP)	-	-	-	-
5	25‡	10	DA	3 (RFP, YFP, mCFP)	+	+	-	-
5	26‡	10	CA	1 (RFP)	+	+	-	-
5	27	10	AA	2 (RFP, mCFP)	-	-	-	-
5	28‡	10	CA	1 (RFP)	+	+	-	-
5	29	10	AA	1 (mCFP)	+	+	-	-
5	30	10	AA	1 (RFP)	+	+	-	-
5	31	10	?	1 (RFP)	-	-	-	-
5	32	10	?	1 (RFP)	-	-	+	-
6	33	10	CA	1 (RFP)	-	-	-	-
6	34	10	CA	1 (mCFP)	-	-	-	-
6	35	10	CA	1 (mCFP)	+	+	-	-
6	36	10	AA	1 (RFP)	-	-	+	-
6	37	10	DA	1 (RFP)§	+	+	-	-
6	38	10	AA	1 (mCFP)	+	+	-	-
6	39	10	DA	1 (RFP)	+	+	-	-
7	40	10	DA	1 (mCFP)	-	-	-	-
7	41‡	10	DA	1 (mCFP)	-	-	-	-
7	42	10	DA	1 (RFP)	-	-	+	-
7	43	10	CA	2 (RFP, YFP)	+	+	-	-
7	44	10	AA	2 (RFP, YFP)	+	+	-	-
7	45	10	?	4 (RFP, YFP, mCFP, nGFP)	+	+	-	-
7	46	10	CA	1 (YFP)	-	-	-	-
7	47	10	AA	2 (RFP, mCFP)	+	+	+	-
7	48	10	?	2 (RFP, nGFP)	-	-	-	-
8	49	10	DA	1 (RFP)	-	-	-	-
8	50	10	DA	1 (mCFP)	+	+	-	-
8	51	10	?	1 (RFP)	-	-	-	-
8	52	10	AA	2 (RFP, mCFP)	-	-	-	-
8	53	10	AA	1 (RFP)	+	+	+	-
8	54	10	DA	2 (RFP, mCFP)	+	+	-	-
8	55	10	CA	2 (RFP, YFP)	+	+	-	-
8	56	10	AA	2 (mCFP, RFP)	-	-	-	-
9	57	10	AA	3 (RFP, YFP, mCFP)	-	-	-	-
9	58	10	AA	1 (RFP)	-	-	-	-
9	59	10	DA	1 (RFP)	-	-	-	-
9	60	10	AA	2 (mCFP, RFP)	+	+	+	-
9	61	10	AA	1 (YFP)	-	-	-	-
9	62	10	AA	1 (RFP)	-	-	-	-

9	63	10	AA	2 (RFP, YFP)	+	+	-	-
9	64	10	DA	1 (RFP)	-	-	-	-
9	65	10	?	3 (mCFP, RFP, YFP)	-	-	-	-
9	66	10	DA	1 (mCFP)	-	-	-	-
10	67	10	DA	1 (YFP)	-	-	-	-
11	68	10	CA	3 (RFP, YFP, mCFP)	-	-	-	-
11	69	10	CA	1 (RFP)	-	-	-	+
12	70	10	AA	2 (RFP, YFP)	-	-	-	+
12	71	10	DA	1 (YFP)	-	-	-	-
13	72	10	CA	2 (RFP, nGFP)	-	-	-	+
13	73‡	10	DA	2 (RFP, mCFP)	-	-	-	+
13	74	10	AA	1 (RFP)	-	-	-	+
13	75	10	DA	2 (YFP, nGFP)	-	-	-	+
14	76	10	AA	1 (RFP)	-	-	-	+
14	77	10	CA	2 (RFP, YFP)	-	-	-	-
14	78	10	CA	3 (mCFP, RFP, YFP)	-	-	-	+
15	79	10	AA	1 (RFP)	-	-	-	-
15	80‡	10	CA	2 (RFP, mCFP)	-	-	-	+
15	81	10	CA	1 (RFP)	-	-	-	-
16	82	10	CA	1 (YFP)	-	-	-	-
17	83	1	AA	1 (RFP)	-	-	-	-
17	84	1	AA	2 (RFP, YFP)	-	-	-	-
18	85	1	AA	1 (RFP)	-	-	-	-
18	86	1	CA	1 (RFP)	-	-	-	-
18	87	1	DA	1 (YFP)	-	-	-	-
19	88	1	AA	1 (RFP)	-	-	-	-
19	89	1	AA	2 (RFP, YFP)	-	-	-	-
19	90	1	CA	1 (RFP)	-	-	-	-
19	91	1	CA	1 (RFP)	-	-	-	-
20	92‡	1	DA	1 (RFP)	-	-	-	-
20	93‡	1	DA	2 (RFP, YFP)	-	-	-	-
20	94	1	CA	1 (RFP)	-	-	-	-
20	95	1	AA	1 (RFP)	-	-	-	-
21	96	1	AA	1 (RFP)	-	-	-	-

* AA: ascending aorta or aortic arch. DA: descending aorta. CA: carotid artery or brachiocephalic artery. Plaques at borders between vascular regions are indicated with a question mark.

† DS: double staining for aSma and Mac3. Only done in plaques that did not contain nGFP or YFP positive cells.

‡ Displayed in figure (Plaque #1: Online Figure VI, C; #8: Online Figure VI, B; #9: Online Figure VI, D; ; #11: Online Figure VI, A, Online Video I; ; #23: Figure 4B; ; #25: Figure 2C; #26: Figure 3AB, Online Figure IV; ; #28: Figure 5AB; ; #41: Figure 2B; ; #73: Online Figure XIIIA; ; #80: Online Figure XIII, B; #92,93: Online Figure VII).

§ One YFP-expressing cell was observed in this plaque.

Online Table IV: Experimental animals used for carotid ligation surgery

Animal no.	Tamoxifen dose (mg)	Back-crosses*	nGFP	YFP	RFP	mCFP	Confetti patches	Measured patches†	Patch size (cell number)	Remodeled area (μm)
1	10	1	3	6	7	4	20	17	48, 52, 149, 156, 227, 375, 425, 522, 585, 872, 950, 981, 1040, 1990, 2290, 3140, 3450	4517
2‡	10	1	1	2	1	3	7	7	171, 409, 666, 849, 940, 1470, 3610	2391
3	10	1	1	1	0	1	3	1	178	1301
4	10	1	0	2	2	0	4	4	174, 332, 415, 508	1878
5	10	1	0	1	1	1	3	3	66, 360, 3090	1276
6	10	1	0	1	2	0	3	3	125, 151, 329	1130
7	10	1	0	2	1	1	4	3	128, 234, 7480	1609
8	10	1	0	3	3	1	7	3	52, 149, 446	2128
9	10	1	0	2	2	2	6	3	125, 805, 1495	1345
10	10	1	0	2	1	1	4	3	96, 115, 1490	1503
11‡	10	6	0	1	1	1	3	3	565, 612, 3200	1994
12	10	6	0	1	1	1	3	2	1097, 2827	2816
13	1	6	0	3	1	2	6	4	35, 63, 689, 1073	NA
14	1	5	2	2	2	0	6	6	50, 55, 81, 98, 148, 225	NA
15	1	6	0	1	1	0	2	2	9, 64	NA
16	1	6	0	1	1	1	3	3	24, 153, 410	NA
17	0.1	1	0	0	0	0	0	0	NA	NA
18	0.1	1	0	1	0	1	2	2	45, 170	NA
19	0.1	1	0	0	0	0	0	0	NA	NA
20‡	0.1	1	0	2	1	0	3	3	65, 192, 749	NA
21	0.1	1	0	0	0	0	0	0	NA	NA
22	0.1	5	0	1	0	0	1	1	32	NA
23	0.1	6	0	1	1	0	2	2	59, 592	NA

* Number of backcrosses to C57Bl/6.
† A small subset of patches was not quantified due to technical issues, such as high autofluorescence, which impeded surface modeling in Imaris.
‡ Displayed in figure (Animal #2: Figure 6BC, Online Figure XII, i; #11: Figure 7AB, Online Figure X, Online Figure XI; #20: Figure 6E, Online Figure XII, ii).
NA, not applicable.

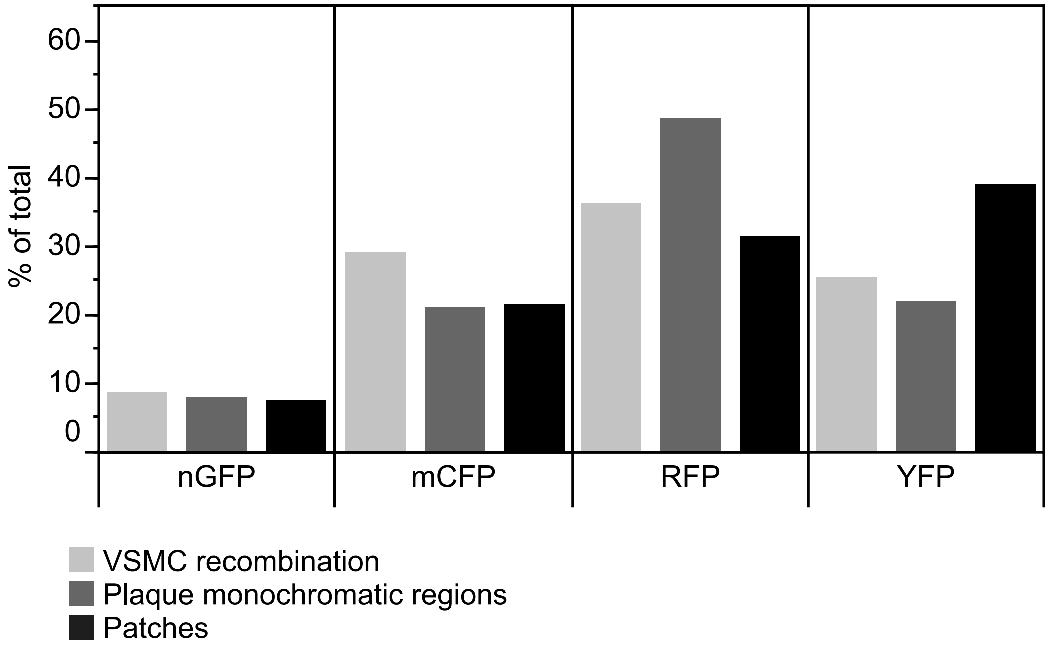

Online Figure I: Frequency of Confetti reporter colors
Bar chart showing the proportions of each of the Confetti colors in VSMCs directly after recombination (light gray), in monochromatic regions within atherosclerotic plaques (dark gray) and in carotid ligation-induced neointimal patches (black). All data is from high density-labeled animals (10x 1 mg tamoxifen

A

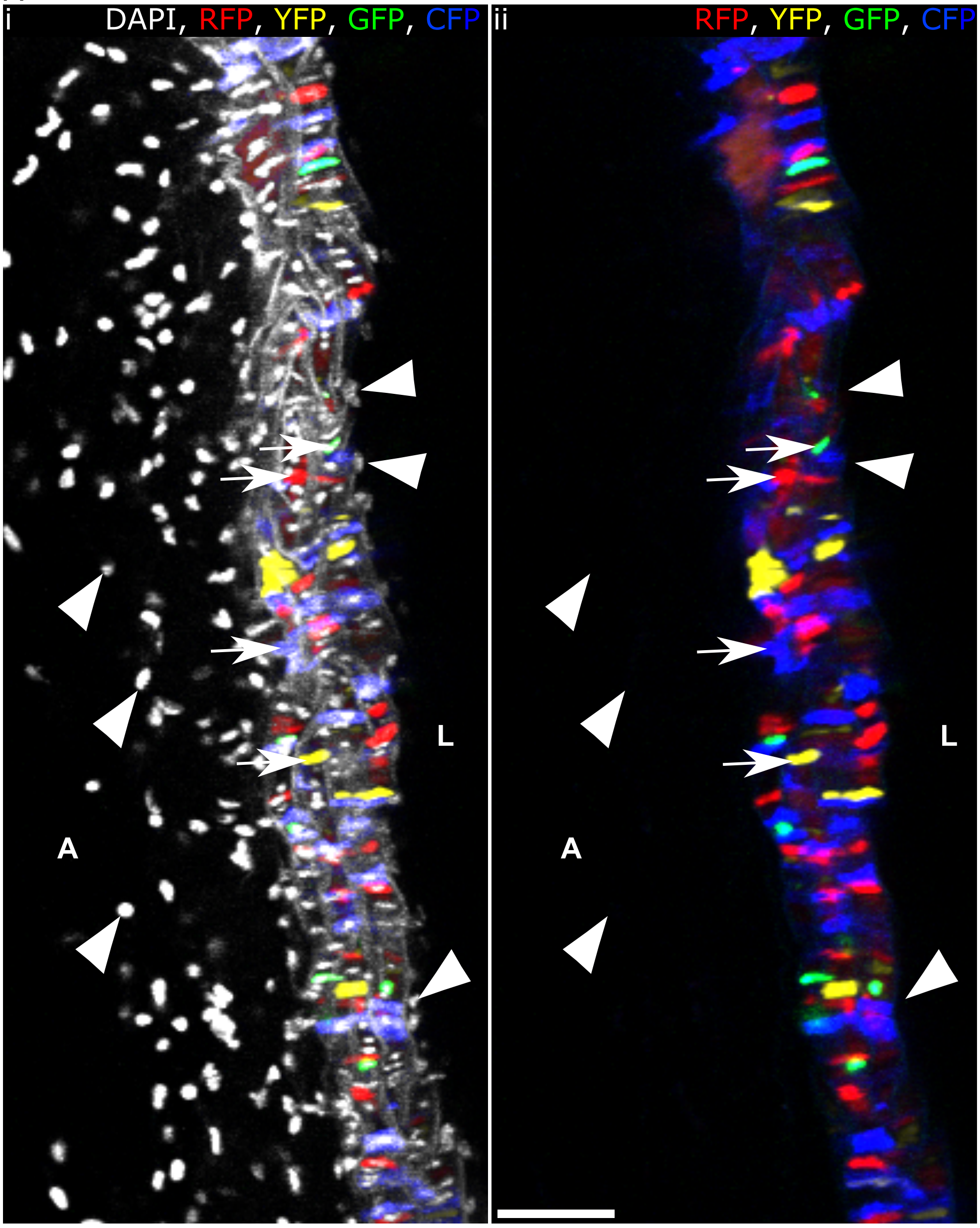

Online Figure II: Mosaic labeling of medial VSMCs

A, Longitudinal cross section of a whole mount carotid artery from a Confetti animal labeled at high density (10x 1 mg tamoxifen). Signals for fluorescent proteins are shown with (**i**) and without (**ii**) nuclear DAPI (white). Arrows point to labeled VSMCs within the medial layer, arrow heads point to unlabeled cells within the endothelium and adventitia. The luminal side is denoted as **"L"** and the adventitial side marked **"A"**. Scale bar is 50 µm.

A

Mouse no.	ID	Sample	Proportion of positive events	Number of singlet events
1	Wild type	Peripheral blood	0.0006	>25000
2	Wild type		0.0003	>65000
3	Wild type		0.00007	>65000
4	Confetti+		0.0001	>65000
5	Confetti+		0.00009	>65000
6	Confetti+		0.0001	>65000
1	Wild type	Bone marrow	0.00002	>400000
2	Wild type		0.00001	>400000
3	Wild type		0.00007	>200000
4	Confetti+		0.00001	>400000
5	Confetti+		0	>400000
6	Confetti+		0.00002	>400000
1	Wild type	Aorta	0.0002	>9000
2	Wild type		0.0001	>30000
3	Wild type		0.0005	>30000
4	Confetti+		0.62	>30000
5	Confetti+		0.63	>30000
6	Confetti+		0.67	>30000

B

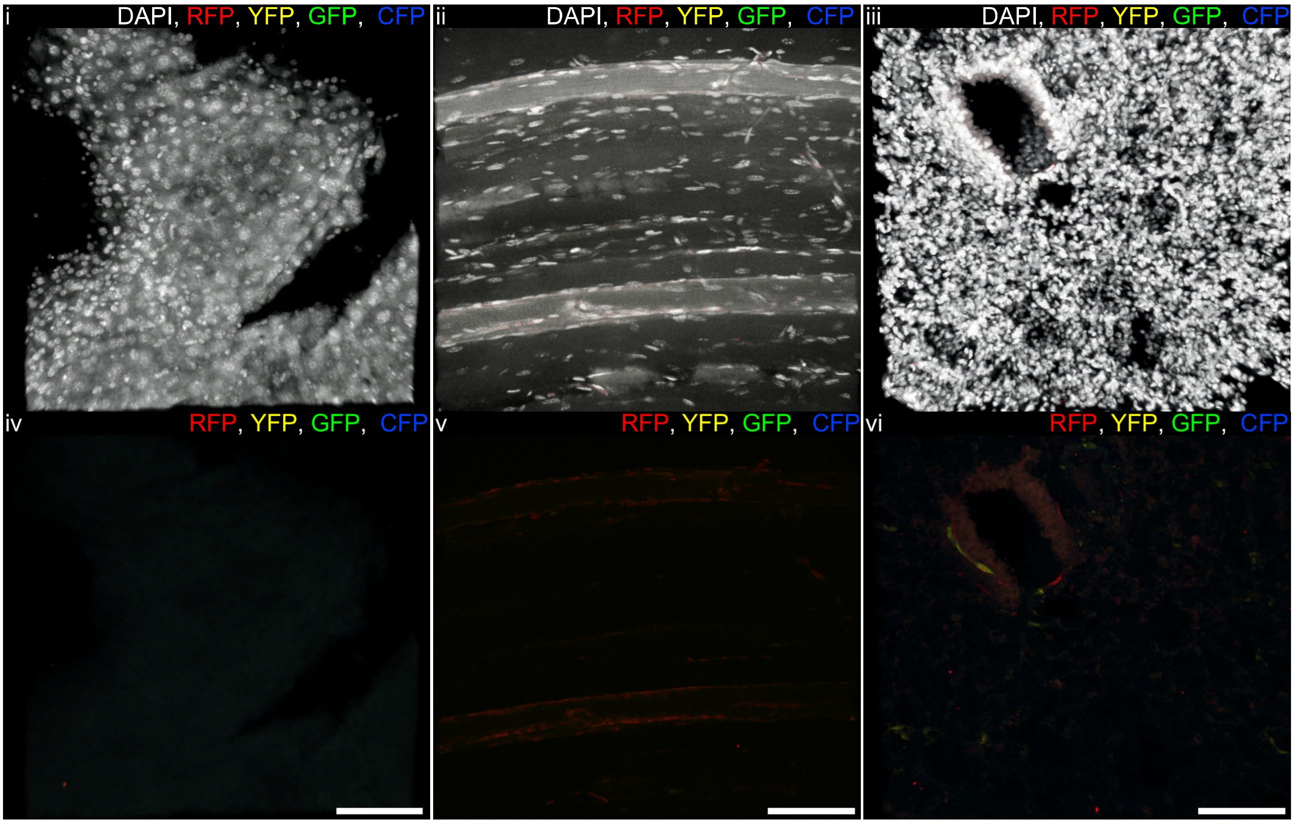

Online Figure III: Specific labeling of VSMCs in Rosa26-Confetti+, Myh11-CreERt2+ animals

A, Flow cytometry analysis demonstrating background levels of fluorescence in bone marrow and peripheral blood from non-tamoxifen treated wild type animals and experimental Rosa26-Confetti+, Myh11-CreERt2+ animals labeled at high density (10x 1 mg tamoxifen). Samples were gated for live cells (FSC-A, SSC-A) and singlets (FSC-A, FSC-H). Negative cells were identified based on gates set on VSMCs from wild type and Confetti animals. **B,** Confocal microscopy images showing absence of recombination in liver (**i, iv**), skeletal muscle (**ii, v**) and lung (**iii, vi**) in Confetti animals after tamoxifen treatment (10x 1 mg). Note, that labeled cells in (**vi**) correspond to VSMCs lining a blood vessel. Signals for fluorescent proteins are shown with (**i, ii, iii**) and without nuclear DAPI staining (white, **iv, v, vi**). Scale bars are 100 µm.

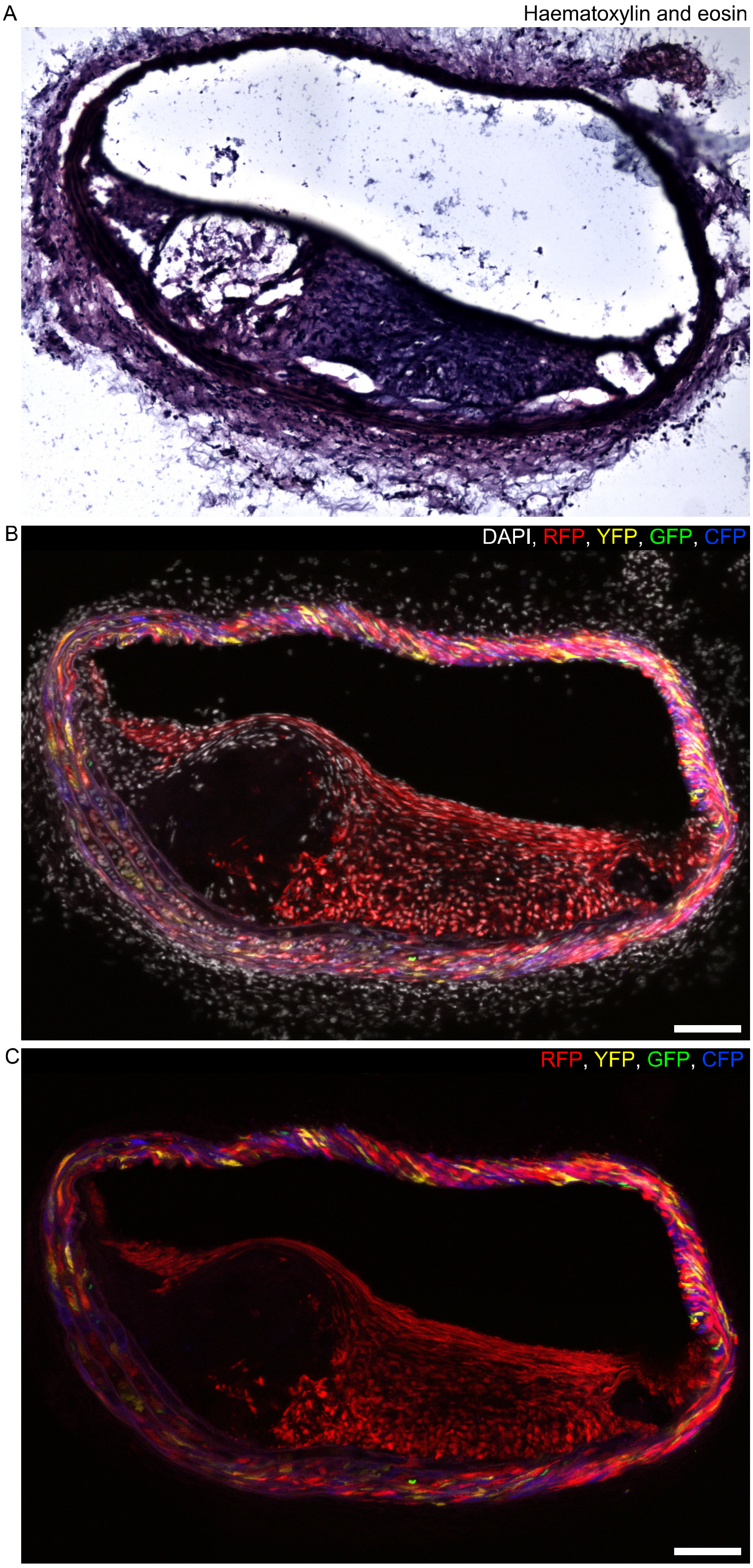

Online Figure IV: Histological analysis of monochromatic plaque region
A, Haematoxylin and eosin staining of 20 µm cryosections from HFD treated Confetti animal labeled at high density (10x 1 mg tamoxifen). For comparison, the signals from fluorescent proteins in an adjacent section are shown with (**B**) and without nuclear DAPI staining (white, **C**). Scale bars are 100 µm.

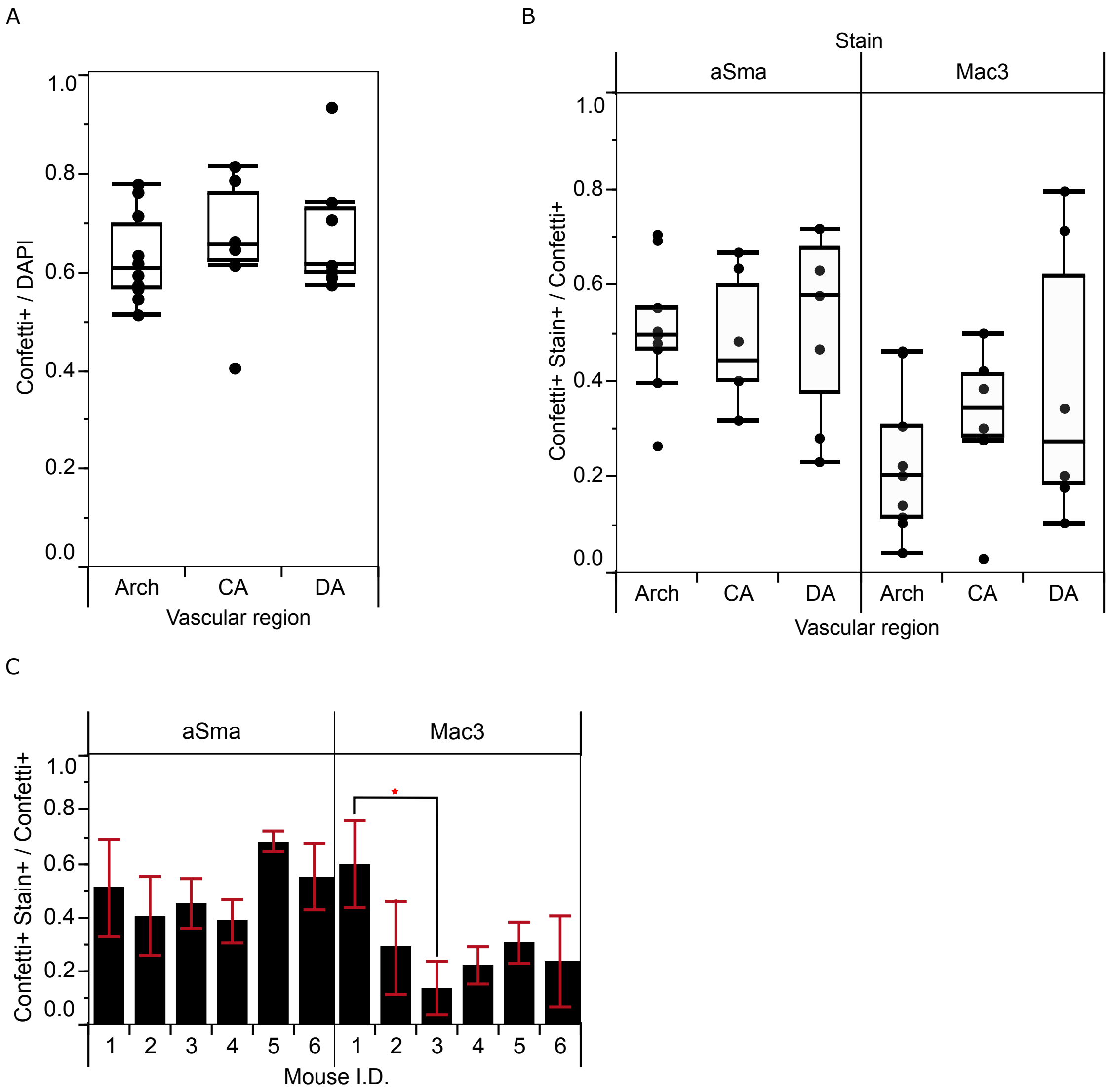

Online Figure V: Proportion of plaque cells expressing the Confetti reporter
A, Box plot quantifying the proportion of all cells (DAPI) within plaques from different regions of the vasculature which express the Confetti reporter (Confetti+). **B, C,** Box plots showing the proportion of cells expressing the Confetti reporter (Confetti+), which were stained for either aSma or Mac3 (Confetti+ Stain+) within plaques from different regions of the vasculature (**B**) or different animals (**C**). Red stars indicate a significant difference based on a two-way ANOVA, $p<0.05$, data from 23 plaques from 6 animals labeled at high density (10x 1 mg tamoxifen). Arch: aortic arch, DA: descending aorta, CA: carotid artery.

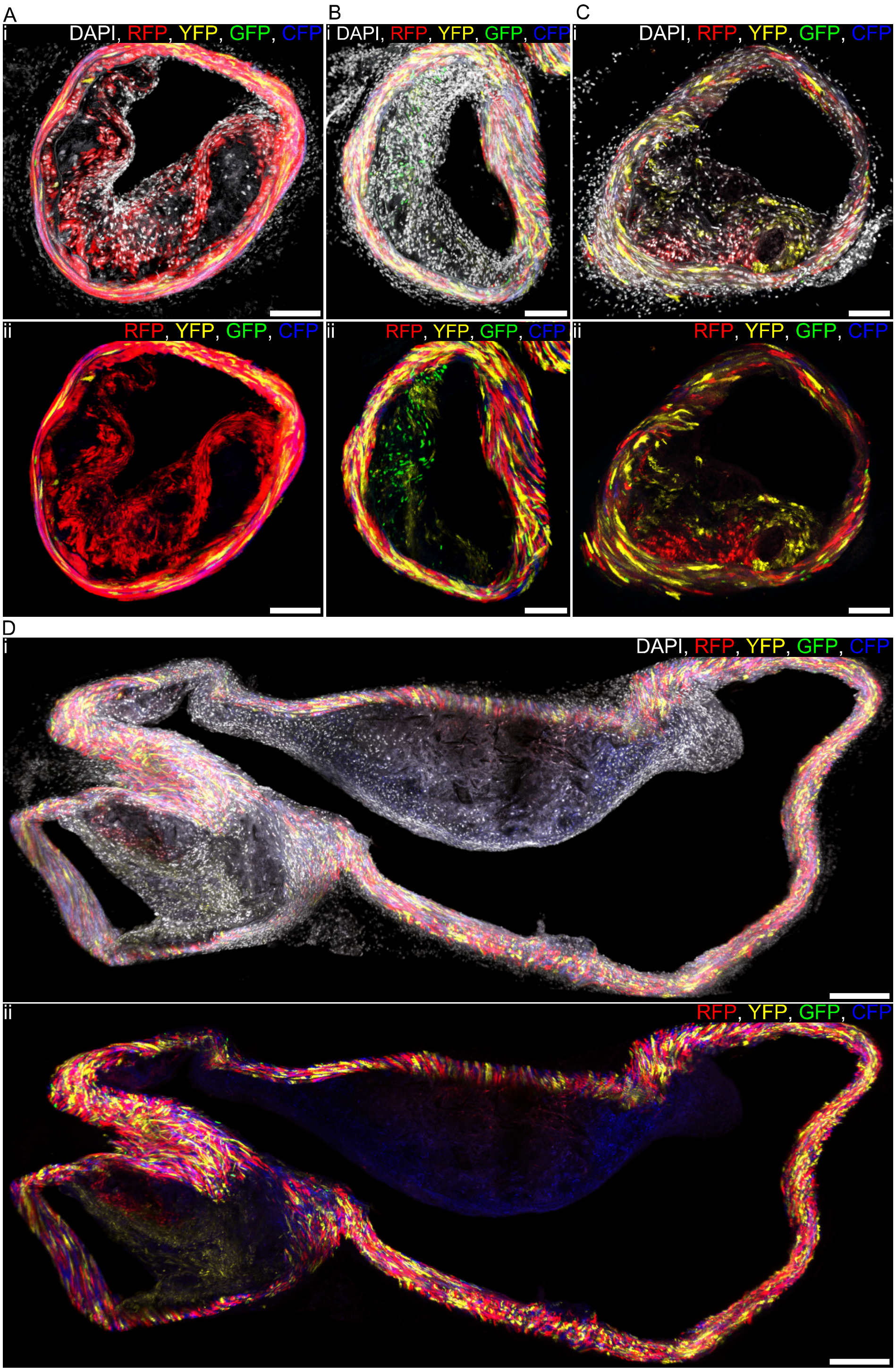

Online Figure VI: Clonal VSMC expansion in atherosclerotic plaques
Examples of monochromatic plaques (**A**) and plaques containing VSMC-derived cells of more than one color (**B-D**). **B,** example of a plaque containing two intermingled clones (yellow and green). The section shown in panel (**D**) is cut through the top of the aortic arch and includes a portion of the right carotid artery. Signals from fluorescent proteins in 50-100 µm vibratome sections are shown with (**i**) and without nuclear DAPI staining (white, **ii**). All plaques are from animals labeled at high density (10x 1 mg tamoxifen). Scale bars are 100 (**A-C**) and 200 µm (**D**). A video of the plaque shown in panel **A** is provided as Supplemental Online Video I.

A

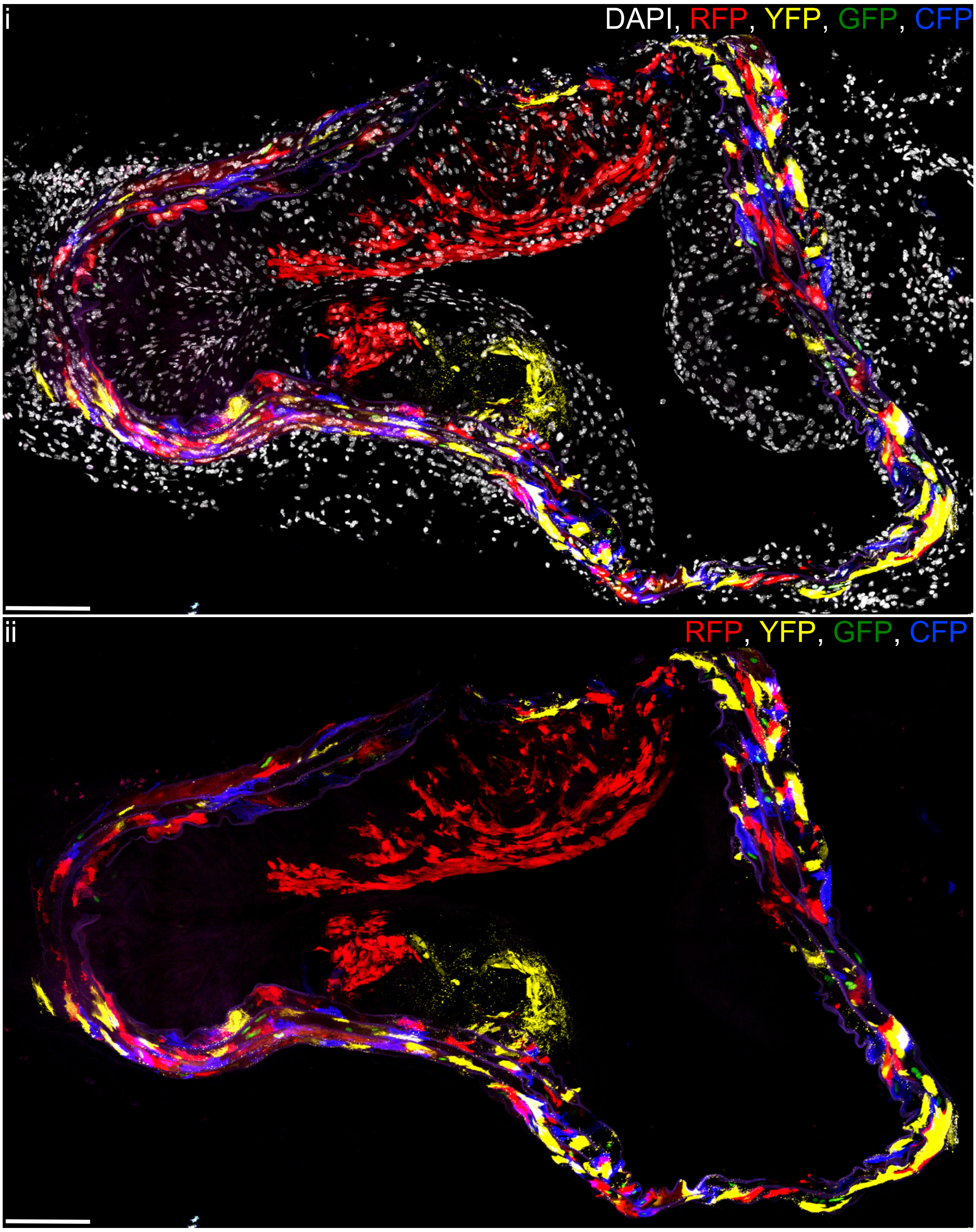

Online Figure VII: Animals labeled at medium density develop plaques with large monochromatic regions, which span both the cap and core

A, An atherosclerotic plaque from a medium density-labeled (1x 1 mg tamoxifen) Confetti animal following 16 weeks of high fat diet. Signals from fluorescent proteins are shown with (**i**) and without (**ii**) nuclear DAPI (white). Scale bars are 100 µm.

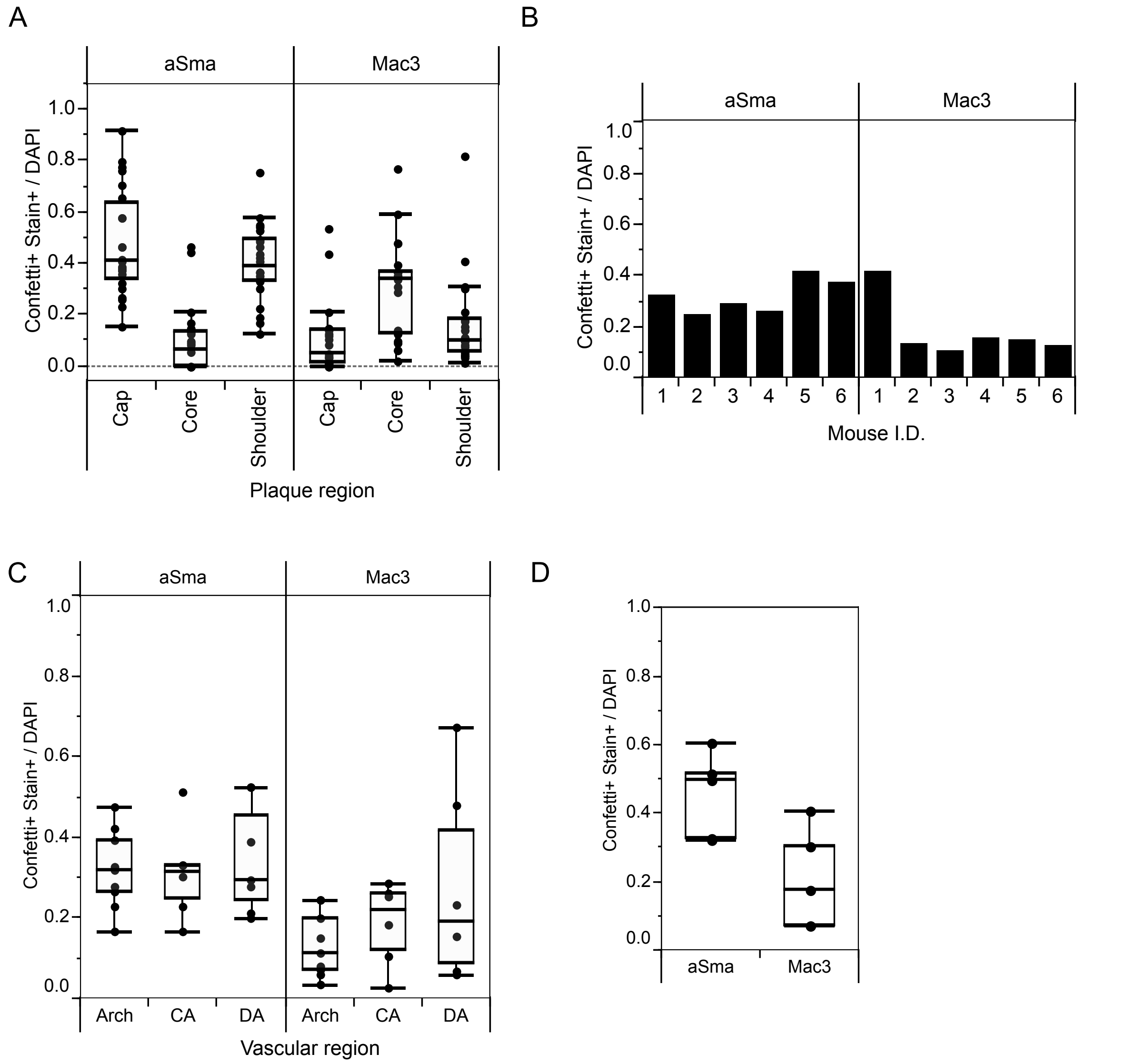

Online Figure VIII: Proportion of all cells within atheroscleotic plaques and injury-induced neointima which are VSMC-derived cells and express phenotype marker proteins

A-C, Box plots displaying the proportion of all plaque cells (DAPI) co-expressing the Confetti reporter and either aSma or Mac3 (Confetti+ Stain+) according to plaque region (**A**), individual animals (**B**) and vascular region (**C**) in high density-labeled (10x 1 mg tamoxifen) Rosa26-Confetti+, Myh11-CreERt2+, ApoE-/- animals after high fat diet. **D,** Proportion of total number of cells (DAPI) which co-express the Confetti reporter and either aSma or Mac3 (Confetti+ Stain+) in carotid ligation-induced neointima from high density-labeled (10x 1 mg tamoxifen) Rosa26-Confetti+, Myh11-CreERt2+ animals 28 days post-surgery

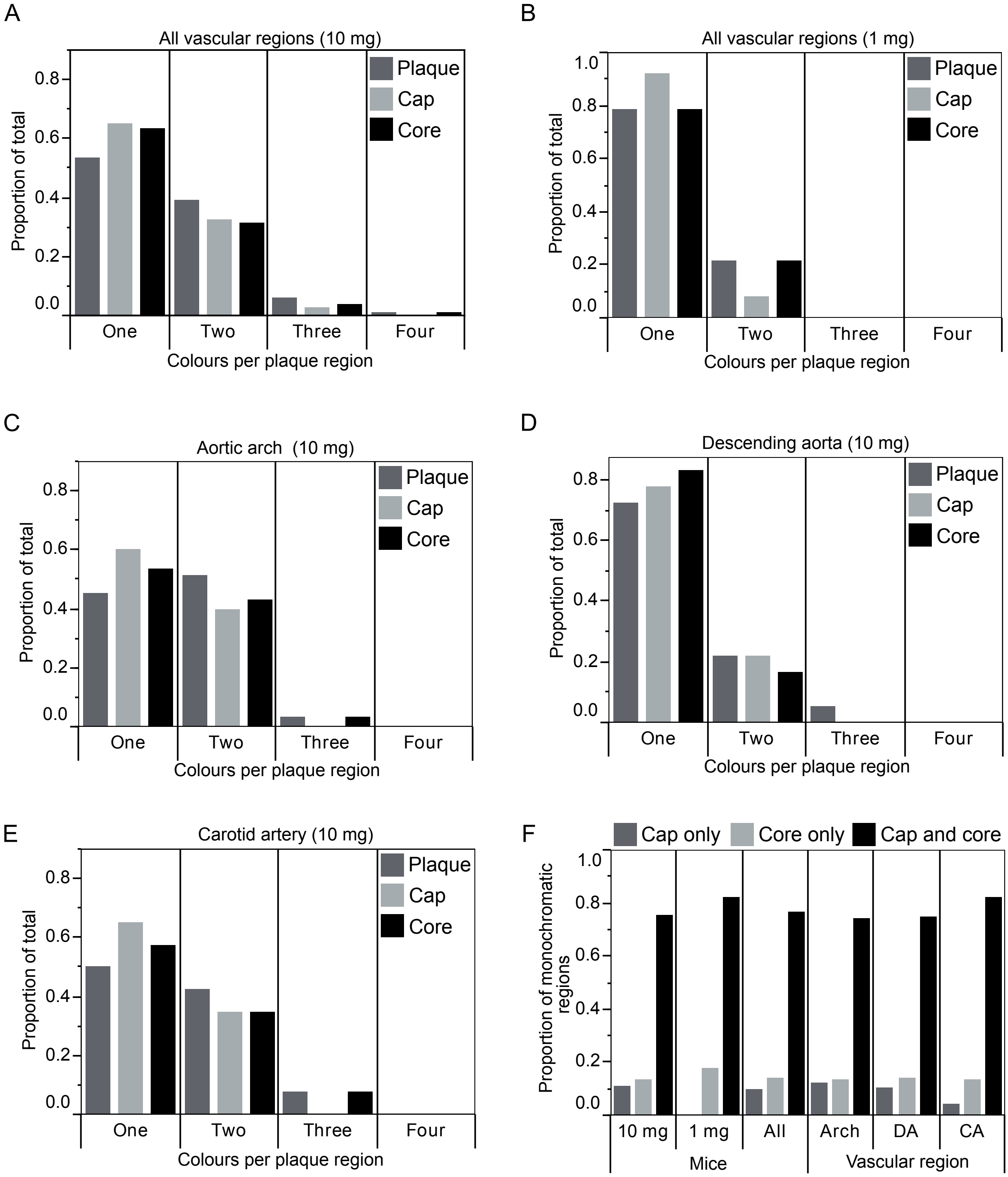

Online Figure IX: Number and distribution of Confetti colors within atherosclerotic plaques

A-E, Bar charts showing the proportion of plaques in high density (10x 1 mg tamoxifen, **A, C-E**) and medium density (1x 1 mg tamoxifen, **B**) labeled animals with one, two, three or four colors. Bars represent the entire plaque (dark gray), the cap (light gray) or the core of the plaque (black). **C, D, E,** Data is shown for plaques in different vascular regions (**C,** Aortic Arch; **D,** Descending aorta; **E,** Carotid artery). **F,** Bar chart showing the proportion of monochromatic regions within plaques which occupy the plaque cap only (dark gray), core only (light gray) or both the core and cap (black). Data is shown for high density (10x 1 mg), low density-labeled (1x 1 mg) or all animals at the left, and stratified according to vascular regions on the right. Arch: aortic arch, DA: descending aorta, CA: carotid artery.

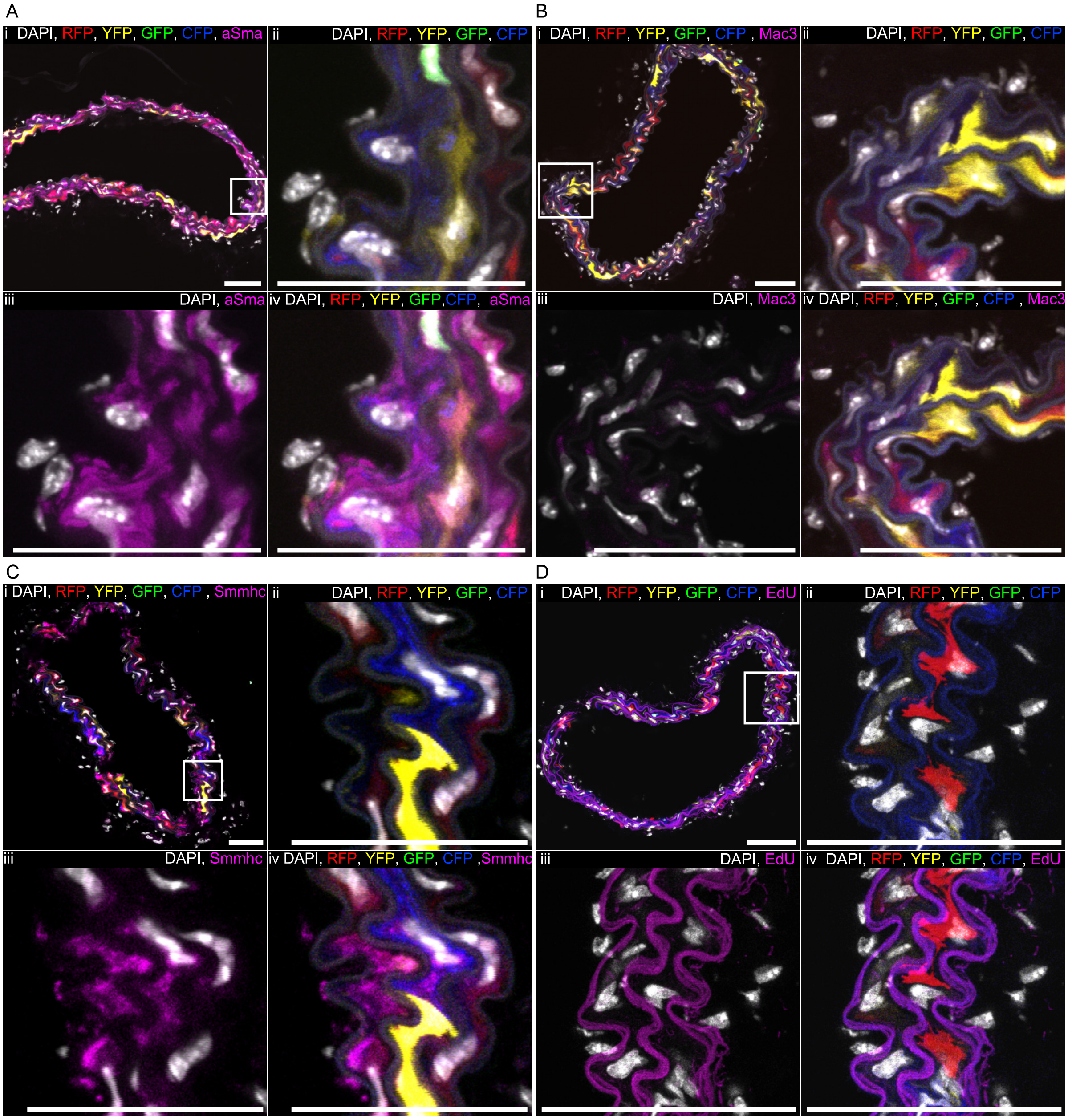

Online Figure X: Right carotid artery immunostaining controls

A-D, Single confocal scans from Z-stacks of 14 μm cryosections from the control right carotid artery of high density-labeled (10x 1 mg tamoxifen) animal 28 days after ligation of the left carotid artery stained for aSma (**A**), Mac-3 (**B**), Smmhc/Myh11 (**C**) or EdU (**D**). For each staining, (**ii-iv**) are zoomed images of the regions outlined in (**i**). Signals for fluorescent proteins, DAPI (white), aSma (magenta), Mac3 (magenta), EdU (magenta), and Smmhc (magenta) are shown as indicated on each image. Scale bars are 50μm.

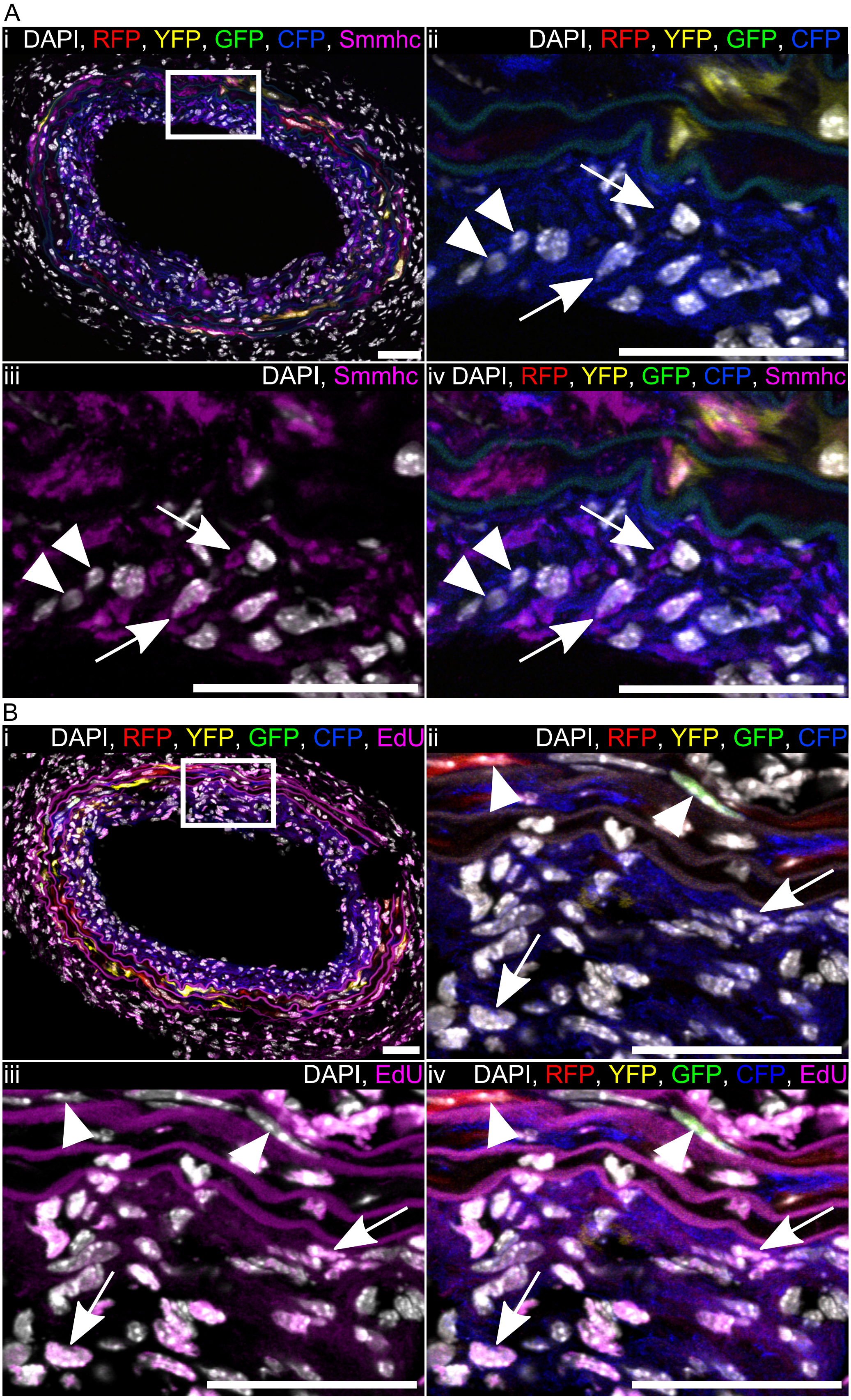

Online Figure XI: Smmhc and EdU staining in carotid ligation-induced neointima
A, B, Single confocal scans from Z-stacks of 14 µm cryosections from the left carotid artery 28 days post-ligation in a high density-labeled animal (10x 1 mg tamoxifen) stained for Smmhc (**A**) or EdU (**B**). For each staining, the region outlined in (**i**) is magnified in (**ii-iv**). Arrows point to cells co-expressing the Confetti reporter and Smmhc (**A**) or EdU (**B**), whereas arrow heads point to cells that express the Confetti reporter, but do not stain for the respective marker. Signals for fluorescent proteins, DAPI (white), Smmhc (magenta) and EdU (magenta) are shown as indicated on each image. Scale bars are 50 µm.

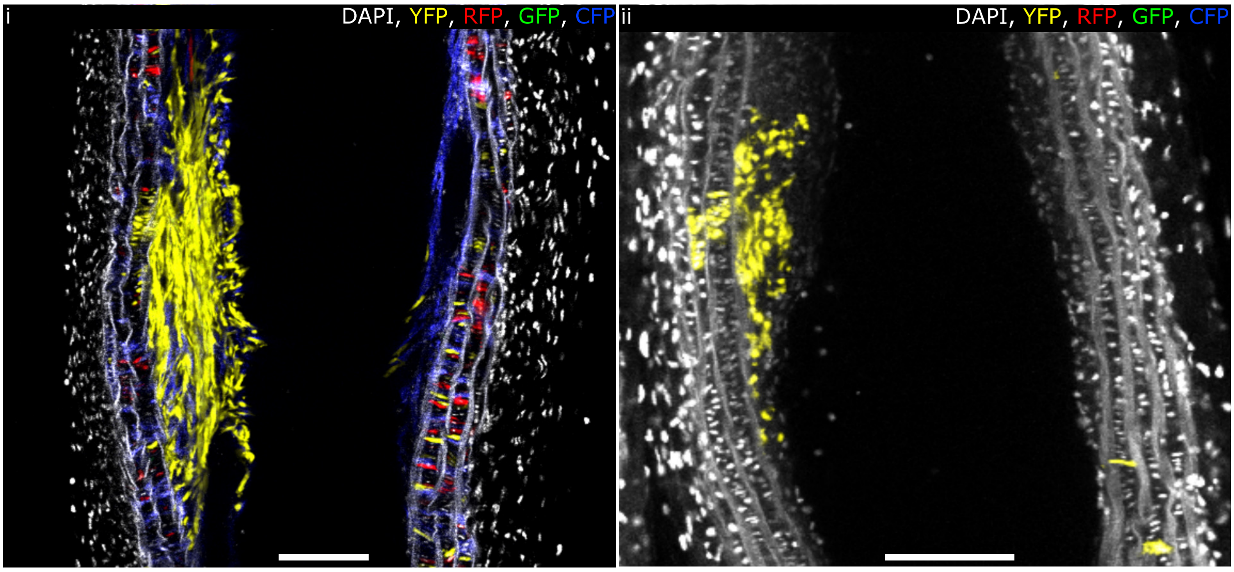

Online Figure XII: Clonal patches span both media and neointima
Longitudinal cross section of the left carotid artery from animals labeled at high density (10x 1 mg tamoxifen, **i**) or low density (1x 0.1 mg tamoxifen, **ii**), which were analyzed 28 days post-surgery, showing that individual VSMC-derived clones are “anchored” in the medial layer. A single scan of a confocal Z-stack, which intersects the middle of the vessel, is shown. Signals for fluorescent proteins and DAPI (white) are shown. Scale bars are 100 μm.

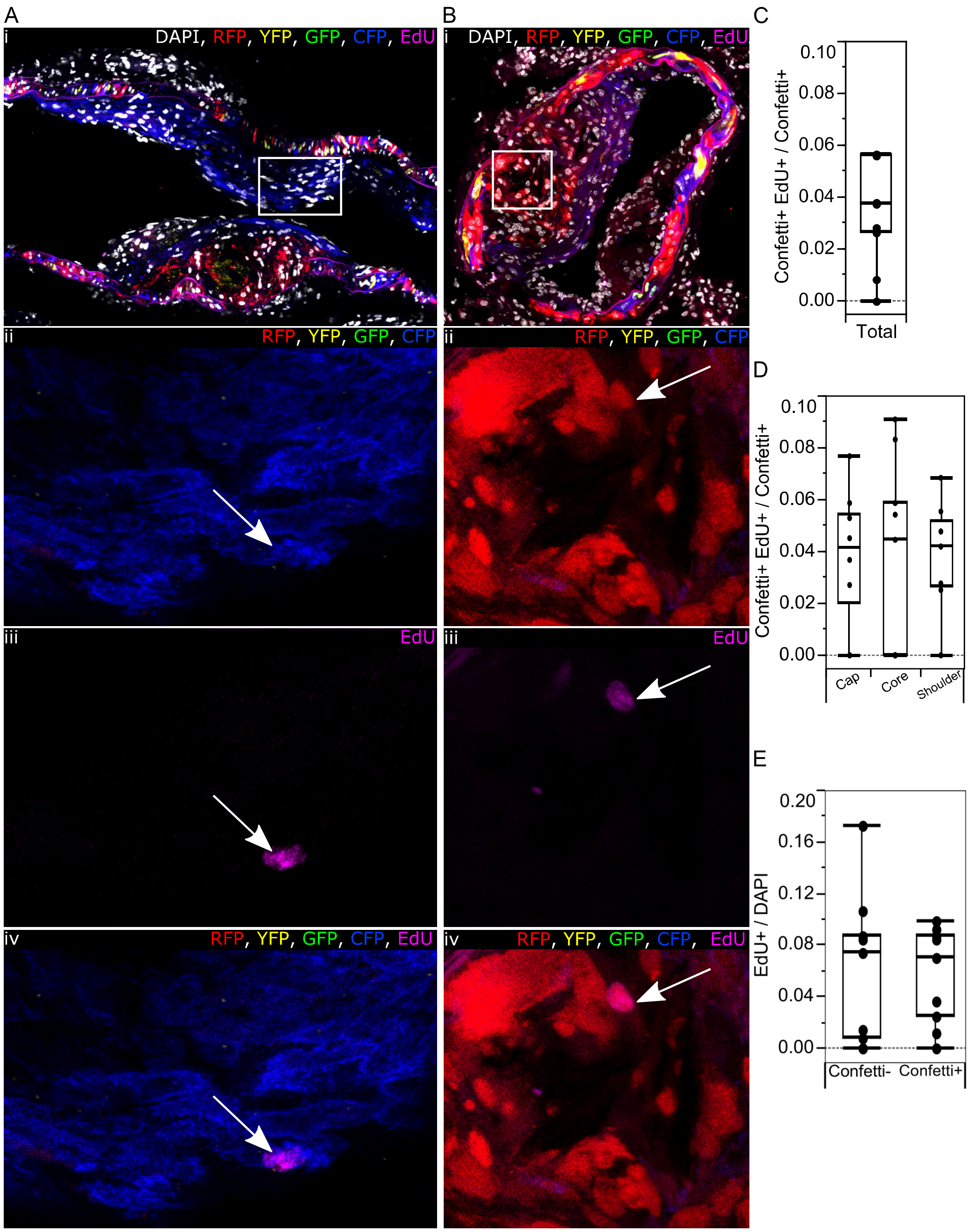

Online Figure XIII: Proliferative VSMC-derived cells detected within both the core and cap of atherosclerotic plaques

A, B, Cryosections of atherosclerotic plaques from Confetti animals subjected to 16 weeks of high fat diet and injected with EdU 2 hours before analysis. The regions outlined in (**i**) are magnified in (**ii-iv**). Arrows point to EdU-positive cells expressing the Confetti reporter in the cap (**A**) or core region (**B**). Signals for fluorescent proteins, DAPI (white) and EdU (magenta) are shown as indicated on each image. **C, D,** Box plot showing the proportion of cells expressing the Confetti reporter (Confetti+) which are EdU+ (Confetti+ EdU+) displayed for all areas analyzed (**C**) and separately for the plaque cap, core and shoulder regions (**D**). **E,** Box plot showing the proportion of all cells (DAPI) which are EdU+. Data in (**C-E**) is from 7 plaques from 6 animals. All data is from high density-labeled animals.
