# Supplementary figures and images for: Extensive Proliferation of a Subset of Differentiated, yet Plastic, Medial Vascular Smooth Muscle Cells Contributes to Neointimal Formation in Mouse Injury and Atherosclerosis Models
Source: Circ Res. 2016 Sep 28;119(12):1313–23. doi: 10.1161/CIRCRESAHA.116.309799 (PMC5149073; doi:10.1161/CIRCRESAHA.116.309799)

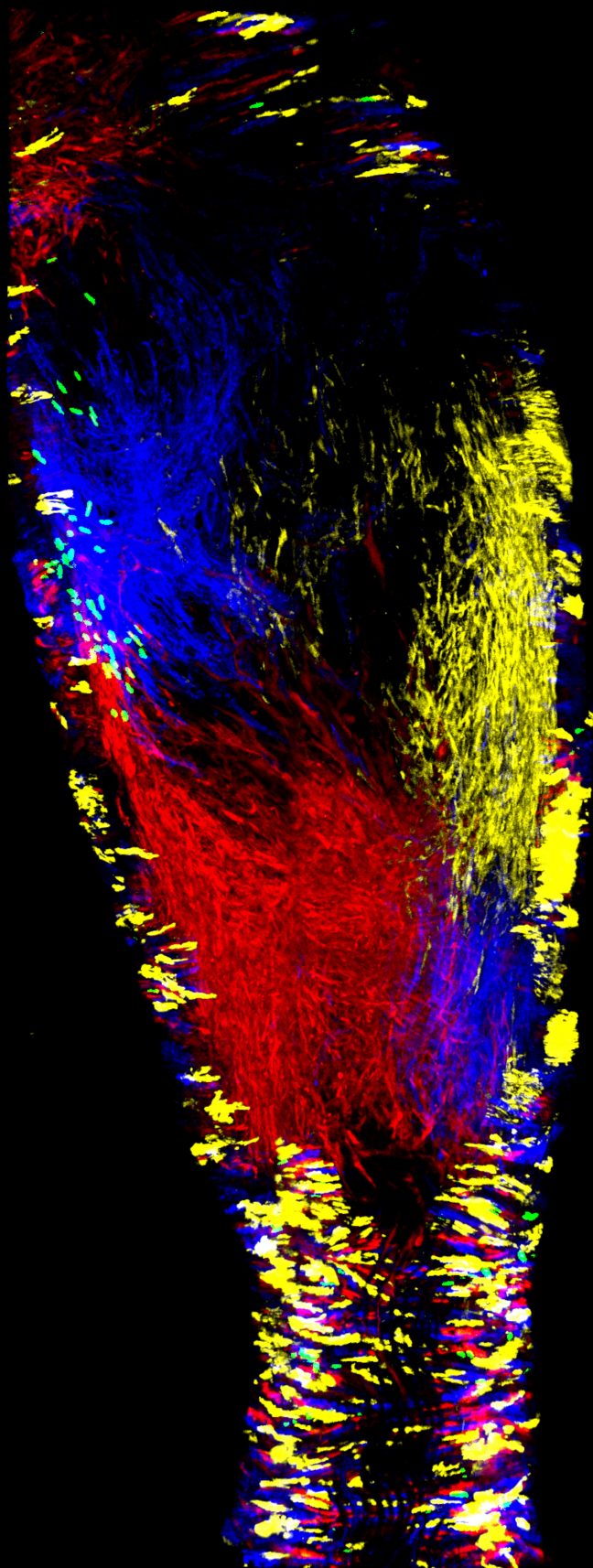

Supplement: Supplementary file 3 [file res-119-1313-s003.pdf]

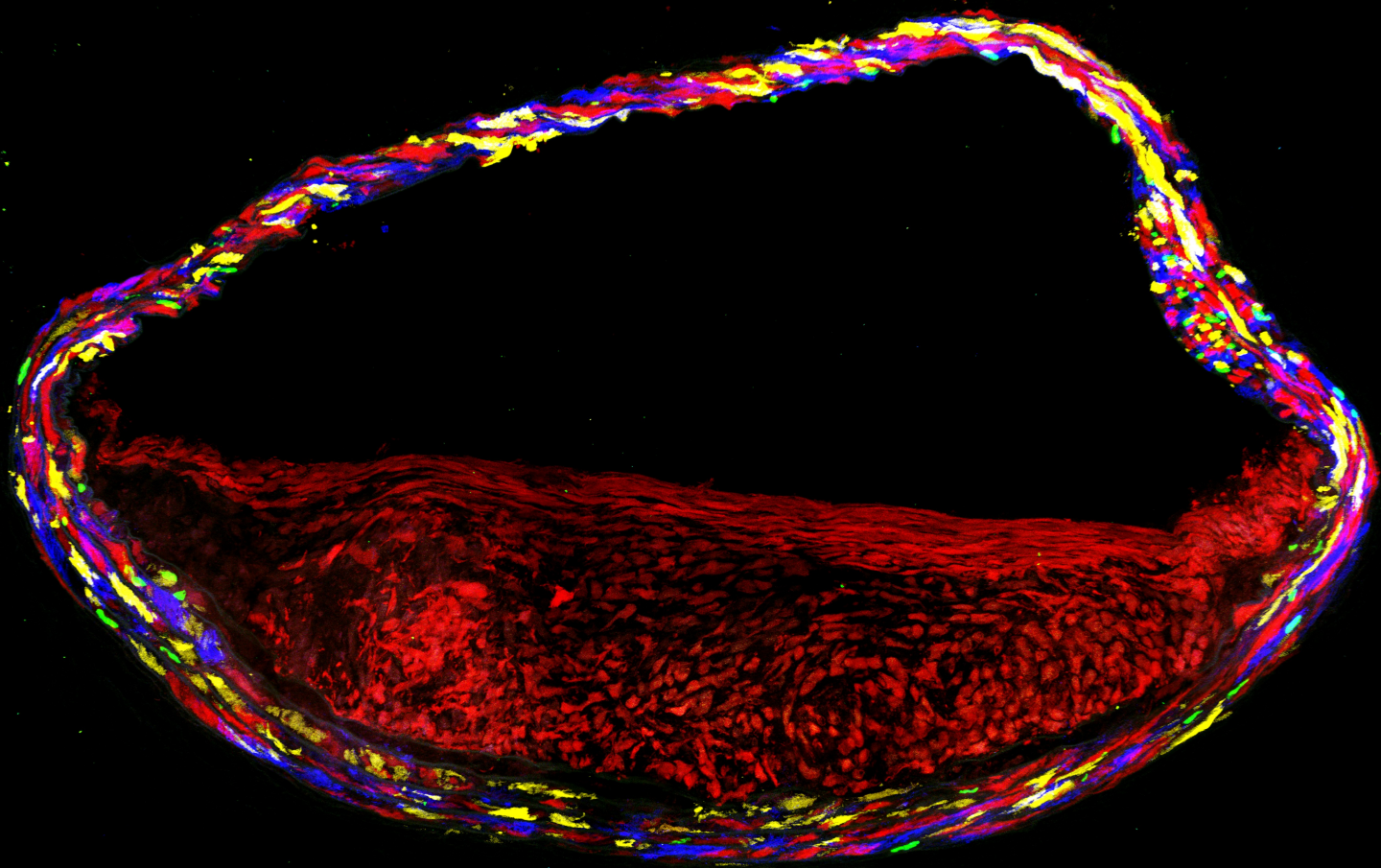

Supplement: Supplementary file 4 [file res-119-1313-s004.pdf]
