# Supplementary material for: Extensive Proliferation of a Subset of Differentiated, yet Plastic, Medial Vascular Smooth Muscle Cells Contributes to Neointimal Formation in Mouse Injury and Atherosclerosis Models
Source: Circ Res. 2016 Sep 28;119(12):1313–23. doi: 10.1161/CIRCRESAHA.116.309799 (PMC5149073; doi:10.1161/CIRCRESAHA.116.309799)
Supplement: Supplementary file 5 [file res-119-1313-s005.pdf]

Cover Image 1:

Maximal projection of three central scans (7 μm apart) of a confocal Z-stack of a whole mounted carotid artery 28 days post carotid artery ligation surgery. Multi-color lineage labeling of mature vascular smooth muscle cells (VSMCs) in Myh11-CreERt2; Rosa26-Confetti mouse reveals that a low proportion of VSMCs expand to contribute to the neointima in arteries following injury. This is demonstrated by observation of large contiguous monochromatic neointimal 'patches'.

Cover Image 2:

Confocal micrograph of an atherosclerotic plaque within a carotid artery (20 μm section). Multi-color lineage labeling of mature vascular smooth muscle cells (VSMCs) in Myh11-CreERt2; Rosa26-Confetti; ApoE-/- mouse reveals that a low proportion of VSMCs expand to contribute to atherosclerotic plaques following high fat diet for 16 weeks. This is demonstrated by the observation that VSMC-derived cells within the plaque are of a single color in contrast to the medial VSMCs which are mosaically labeled.
